# Impact of drought stress on simultaneously occurring pathogen infection in field-grown chickpea

**DOI:** 10.1038/s41598-019-41463-z

**Published:** 2019-04-03

**Authors:** Ranjita Sinha, Vadivelmurugan Irulappan, Basavaiah Mohan-Raju, Angappan Suganthi, Muthappa Senthil-Kumar

**Affiliations:** 1 National Institute of Plant Genome Research, JNU Campus, Aruna Asaf Ali Marg, New Delhi, 110067 India; 2 Department of Crop Physiology, University of Agricultural Sciences, GKVK, Bangalore, 560065 India; 3 Agricultural research station and Krishi Vigyan Kendra, Tamil Nadu Agricultural University, Virinjipuram, Vellore, 632104 India; 4 Present Address: Tamil Nadu Agricultural University, Coimbatore, 641003 India

## Abstract

Drought stress and pathogen infection simultaneously occur in the field. In this study, the interaction of these two stresses with chickpea, their individual and combined effect and the net impact on plant growth and yield traits were systematically assessed under field and confined pot experiments. The field experiments were conducted for four consecutive years from 2014–15 to 2017–18 at different locations of India. Different irrigation regimes were maintained to impose mild to severe drought stress, and natural incidence of the pathogen was considered as pathogen stress. We observed an increased incidence of fungal diseases namely, dry root rot (DRR) caused by *Rhizoctonia bataticola*, black root rot (BRR) caused by *Fusarium solani* under severe drought stress compared to well-irrigated field condition. Similar to field experiments, pot experiments also showed severe disease symptoms of DRR and BRR in the presence of drought compared to pathogen only stress. Overall, the results from this study not only showed the impact of combined drought and DRR stress but also provided systematic data, first of its kind, for the use of researchers.

## Introduction

Chickpea is mainly cultivated in the arid and semi-arid tropical regions under rain-fed condition^1^. It is susceptible to terminal drought stress due to decreased rainfall and depletion of stored soil moisture towards maturation, and experiences up to 50% yield loss^2–4^. Chickpea is also known to be susceptible to 172 pathogens^5^. Among all pathogen stresses, under the favorable condition, ascochyta blight (AB, *Ascochyta rabiei*), botrytis gray mold (BGM, *Botrytis cinerea*), dry root rot (DRR, *Rhizoctonia bataticola*) and fusarium wilt (FW, *Fusarium oxysporum*) can cause up to 100% destruction to the crop^5^. Moreover, chickpea growing fields are vulnerable to both drought and pathogen stress. For instance, drought-prone areas in central and south India are prone to DRR, collar rot (CR, *Sclerotium rolfsii*), black root rot (BRR, *Fusarium solani*) and ascochyta blight disease as well^6–8^.

Naturally, stresses occur in combination in the field. Drought stress influences the plant interaction with the pathogen when both the stress co-occur^9–14^. Earlier, drought stress has been shown to increase the incidence of DRR^15–17^ and FW^18^ in chickpea under laboratory conditions. On the contrary, incidence of CR^19^ and wet root rot^5^ (WRR, *Rhizoctonia solani*) increases under high soil moisture. These studies indicate that the three-way interaction of plant-drought-pathogen could be positive or negative. Moreover, such interactions are complex under field conditions, and it depends on the soil microbiome-plant phytobiome combination, and weather factors prevailing in the cultivation area^9–11^. These multiple stress interactions can occur in various possible ways, for instance, two stresses can either happen sequentially or else simultaneously^14^.

All these multiple stress interaction possibilities and their outcome are not yet studied and demand a thorough evaluation using well-designed experiments^10,12,14^. Combined drought and pathogen stress are known to alter physio-morphological traits such as photosynthesis, stomatal conductance, and transpiration rate^20^ along with plant growth and root morphology^21,22^. Thus, understanding the actual impact of multiple combined stresses on yield-related factors in field-grown essential crops is also needed. Moreover, data from systematic combined stress studies can be used to predict the occurrence of stresses in the future for various drought-affected regions by simulation modeling to assist in the development of strategies to overcome the combined stress effect^23^.

In this study, we conducted field trials at different research stations located in different agro-climatic regions of India for four consecutive years to study the effect of different drought stress levels namely mild, moderate and severe on disease incidence and the net impact of combined stress on growth and yield. Further, we reconfirmed the outcome of field experiment by replicating pathogen, drought and combined stress treatments in well-planned confined pot experiments in the laboratory.

## Results

### *In silico* and literature analysis showed co-occurrence of drought and pathogen in various chickpea growing regions in India

We obtained the rain-fall data for the year 2010–11 and 2013–14 from Open Government Data platform (Data Gov; https://data.gov.in/) for India and examined the incidences of some of the economically important chickpea diseases such as DRR, BRR, CR and FW in the same area based on literature information^6–8^. We separately plotted the incidence of each disease with the drought occurrence in chickpea growing areas (Supplementary Fig. S1A–D). Disease occurrence was found to be predominant in severe drought stress months thus indicating the co-occurrence of root rot disease and drought stress in nature (Supplementary Fig. S1). However, until now, a systematic study to assess the interaction between drought and pathogen at plant interphase and the net impact of these combined stresses in the field has not been conducted. Our study was designed to address this gap.

### Soil moisture measurements confirmed accurate imposition of the different levels of drought stress in the field

The field experiment comprised seven treatments, with three different levels of drought stress (DS; namely mild DS, moderate DS and severe DS), three combined stress treatments (CS; named as mild CS, moderate CS and severe CS, based on corresponding drought stress) and a well-irrigated pathogen treatment, along with well-irrigated control (Supplementary Figs S2, S3, S4 and S5, Supplementary Video S1). Chickpea plants in control and drought stress plots were treated with fungicide to control the pathogen incidence. Drought stress was imposed in both DS and CS plots by increasing the time-gap between the irrigations (see materials and methods). Drought stress levels in field location-1 treatments were measured as relative soil moisture content (Supplementary Figs S6 and S7). Reduction in soil moisture was observed under drought and combined stress treatments compared to control and pathogen only treatments. Soil moisture data were in accordance with the irrigation frequency. Severe DS and severe CS treatment plots, which received lesser number of irrigations, usually had very low soil moisture (except for few days after irrigation; Supplementary Figs S6 and S7). This data on low soil moisture status depicted the imposition of desired drought stress.

Drought stress in field location-2 treatment plots was measured as soil water potential (Ψ_w_) (Supplementary Fig. S8A,B) and crop canopy temperature (Supplementary Fig. S8C–E). Crop canopy temperature is one of the indirect methods to measure drought stress level in plants. Canopy temperature of the crop increases under drought stress due to decreased transpiration rate as stomata close under drought to avoid water loss. High crop canopy temperature around 95 °F was observed in moderate DS, severe DS, and severe CS treatment plots compared to around 92 °F in control and pathogen only treatment plots during 2015–16 (Supplementary Fig. S8C). Similarly, canopy temperature increased from 88 °F in control and pathogen treatment plots to 93 °F in severe DS and 95 °F in severe CS treatment plots in the year 2017–18 (Supplementary Fig. S8D). Overall, both decreased soil water potential and increased canopy temperature confirmed the appropriate imposition of drought stress in the plots.

### Field observations revealed the occurrence of several diseases caused by fungi and viruses

The foliar and root parts of chickpea plants in the treatment plots were examined for the presence of disease symptoms. Majorly, we observed the occurrence of DRR, BRR and virus disease similar to *Beet western yellow virus* (BWYV) in field location-1 and 2 (Supplementary Fig. S9B–F,H). Plants at field location-3 had sclerotinia stem rot caused by *Sclerotinia sclerotiorum* (Supplementary Fig. S9G).

Plants with DRR disease showed symptoms such as dry foliar part and main root without lateral roots. Plants were easy to uproot from the soil due to loss of lateral roots and rotten primary root. The symptomatic plants had dry and brittle root along with the presence of black microsclerotia in the vascular tissue (Supplementary Fig. S9B, Supplementary Video S1). Plants having BRR disease were identified by the yellowing of lower leaves and rotten black roots. Also, white mycelial growth was seen over the root surface of BRR infected plants (Supplementary Fig. S9C, Supplementary Video S1). However, the incidence of CR and FW in the field locations was negligible and found in only a few plots. FW plants showed brown discoloration of vascular tissue at root junction and shoot region (Supplementary Fig. S9D). Plants with CR disease was distinguished by a lesion around the collar region and wilting of the whole plant (Supplementary Fig. S9E). Plants also showed viral disease symptoms like reddening of leaflet margin and shoot branches which were similar to BWYV infection (Supplementary Fig. S9H). Further, DRR and BRR diseases were confirmed by isolation of fungal mycelia from infected roots (Supplementary Fig. S9Biii,Ciii) and confirmation of fungi by PCR and sequencing with universal ITS primers (Supplementary Figs S9I,J).

### Drought stress increased the incidence of BRR and DRR in chickpea

Among the diseases observed in this field study, BRR and DRR were the major diseases especially under severe CS with an average incidence of 25–40% (Tables 1 and 2). Percent disease incidence of BRR was high in severe CS treatment compared to the pathogen and mild CS treatment in field location-1 and field location-2 in all the years studied. BRR incidence in severe CS varied from around 1.5- to 3.7-fold as compared to pathogen treatment and from around 1.2-fold to 2.1-fold compared to mild CS treatment (Table 1). Similarly, moderate CS had increased BRR incidence (1.5 to 3-fold) compared to pathogen treatment. However, no difference in percent disease incidence was observed between moderate and severe CS, as well as pathogen and mild CS treatments (Table 1). BRR disease incidence in control and mild DS treatment plots were very low as chickpea genotype was moderately resistant to the diseases. However, disease incidence in moderate and severe DS could not be controlled despite fungicide treatments. This indicted towards compromised immunity of chickpea under the drought stress. Severe DS had 1.8 to 14-fold higher BRR incidence compared to control and 1.1 to 11-fold higher incidence compared to mild DS. BRR disease incidence was also high in moderate DS compared to control (Table 1). BRR incidence in severe CS was around 1.3- to 1.9-fold higher compared to severe DS in the field location trials (Table 1). However, disease incidence between severe DS and severe CS did not vary in field location-2. This could be probably due to the compromised effect of fungicide under high pathogen load in sick plots. No significant difference in BRR incidence was found between control and pathogen treatments, as well as mild DS and mild CS for 2015–16 and 2016–17 field experiments, which could probably be due to reduced effectiveness of the fungicide in sick plots (Table 1). A high correlation was observed for BRR disease incidence between trials as *r*-values ranged from 0.75 to 0.98 (*p* < 0.05) (Supplementary Table S2). Disease incidence in field location-2 during 2015–16 and 2016–17 were higher in control and pathogen due to high pathogen load in sick plot and conducive environmental conditions (Table 1).

**Table 1:** The incidence of black root rot disease under drought and combined stress treatments in two different locations of field trial from the year 2014 to 2018^*^.

Treatment	2014-15	2015-16		2016-2017		2017-2018	
	Field-1	Field-1	Field-2	Field-1	Field-2	Field-1	Field-2
Control	4.167^a^	1.938^a^	8.571^a^	0.625^a^	35.14^a^	0^a^	11.588^a^
Mild DS	9.167^ab^	2.908^a^	9.524^ab^	1.78^ab^	47.98^bc^	10.041^bc^	14.52^ab^
Moderate DS	10^b^	3.531^a^	9.524^ab^	6.78^bc^	56.48^de^	11.968^cd^	25.906^cd^
Severe DS	14.167^bc^	7.9^c^	10.476^ab^	8.96^c^	63.29^ef^	15.641^de^	31.824^de^
Pathogen	10^b^	3.851^ab^	9.048^ab^	1.11^a^	42.85^ab^	6.2723^b^	19.885^bc^
Mild CS	15.833^c^	6.544^bc^	11.905^ab^	6.81^bc^	54.73^cd^	10.597^bc^	20.56^bc^
Moderate CS	24.167^d^	7.151^c^	12.857^ab^	7.51^c^	65.59^f^	19.111^ef^	27.97^de^
Severe CS	26.667^d^	14.123^d^	16.667^b^	18.6^d^	66.36^f^	20.46^f^	35.203^e^
**Grand mean**	**14.271**	**5.9932**	**11.071**	**6.5243**	**54.057**	**11.761**	**23.433**
**CV**	**16.18**	**19.8**	**41.7**	**35.46**	**8.29**	**15.71**	**17.66**
**LSD at** ***p*** ** < 0.05**	**5.4604**	**2.8066**	**8.0858**	**5.4711**	**7.8507**	**4.3702**	**7.2458**

^*^Data represents percent BRR incidence in chickpea field location 1 and 2. Average of 2-3 block replicates are represented here. RCBD two-way ANOVA was used for the comparison of means of each field experiment and significance was assessed by the least significant difference (LSD at *p* < 0.05) post hoc test. Statistics are performed column-wise. Different letters (a, b, c, d, e, f) in a column denote significant difference in mean at *p* < 0.05. DS = drought stress; CS = combined stress; CV = coefficient of variance; LSD = least significant difference; Field location-1 = NIPGR, New Delhi; Field location-2 = GKVK, Bengaluru. Data shows an increase in the BRR incidence under severe CS and severe DS treatments compared to pathogen treatment and control.

**Table 2:** The impact of drought on the incidence of dry root rot disease in two different locations of field trial from the year 2014 to 2018^*^.

Treatment	2014-15		2015-16		2016-2017		2017-2018	
	Field-1	Field-2	Field-1	Field-2	Field-1	Field-2	Field-1	Field-2
Control	3.75^a^	12.00^a^	0^a^	1.47^a^	0^a^	0^a^	0^a^	4.387^a^
Mild DS	7.083^ab^	NA	4.81^a^	1.84^a^	0^a^	1.5278^a^	0^a^	9.583^ab^
Moderate DS	11.25^bc^	NA	0^a^	2.39^a^	0^a^	2.2276^a^	0^a^	19.558^bc^
Severe DS	14.583^cd^	NA	32.5^c^	7.04^ab^	0^a^	33.815^bc^	4.1729^ab^	40.396^de^
Pathogen	8.75^abc^	12.80^a^	0^a^	1.59^a^	7.605^ab^	4.8544^ab^	1^a^	9.065^ab^
Mild CS	10.833 ^bc^	18.86^a^	0^a^	4.32^ab^	0^a^	7.522^ab^	0^a^	16.854^abc^
Moderate CS	11.667^bc^	30.45^b^	18.119^b^	10.00^bc^	14.95^b^	11.828^ab^	10.157^ab^	28.592^cd^
Severe CS	19.583^d^	36.77^b^	35.294^c^	15.964^c^	42.23^c^	53.23^d^	13.333^b^	42.414^e^
**Grand mean**	**10.937**	**22.184**	**11.34**	**5.5801**	**8.0985**	**14.376**	**3.5829**	**21.356**
**CV**	**41.71**	**18.29**	**22.5**	**64.58**	**53.02**	**88.06**	**132.14**	**35.51**
**LSD at** ***p*** < **0.05**	**6.7093**	**11.268**	**6.0335**	**6.3109**	**10.153**	**29.933**	**11.195**	**13.282**

^*^Data represents percent DRR incidence in chickpea field location 1 and 2. Average of 2–3 block replicates are represented here. RCBD two-way ANOVA was used for the comparison of the mean for each experiment and significance was assessed by the least significant difference (LSD at *p* < 0.05) post hoc test. Statistics are performed column-wise. Different letters (a, b, c, d, e) in a column denote significant difference between mean at *p* < 0.05. DS = drought stress; CS = combined stress; CV = coefficient of variance; LSD = least significant difference; NA = treatments could not be maintained in field experiment thus data is not available. Field location-1 = NIPGR, New Delhi; Field location-2 = GKVK, Bengaluru. Data shows an increase in the DRR incidence under severe CS treatment compared to pathogen treatment.

Root morphology of plants uprooted from severe CS treatment plots showed heightened BRR disease symptoms compared to plants from pathogen treatment (Supplementary Figs S10 and S11, Supplementary Video S1). The plant from severe DS and severe CS had low foliar biomass compared to control in both the field locations (Supplementary Figs S10 and S11, Supplementary Video S1).

Similar to BRR, percent disease incidence of DRR increased in the severe CS compared to the pathogen and mild CS stress treatments in all the field locations and through all the years. DRR incidence in severe CS was 2- to 11-fold higher compared to pathogen treatment, and 1.8- to 7-fold higher compared to mild CS (Table 2). Severe CS also showed high DRR incidence over moderate CS in field location-1 trials and few field location-2 trials. No significant difference was observed in DRR incidence between pathogen only and mild CS. DRR incidence was either absent or very low in control, mild DS and moderate DS plots. However, it was very high in severe DS compared to control and mild DS. There was no difference in DRR incidence between control, mild DS, pathogen and mild CS as the incidence was very low in all these treatments. Also, DRR incidence did not differ between severe DS and severe CS as even severe DS had high DRR incidence (Table 2). The pattern of DRR incidence between different field trials was highly correlated (most of the *r*-values ranged from 0.72 to 0.99, *p* < 0.05; Supplementary Table S3).

Moreover, root symptoms also confirmed the increased susceptibility of plants facing severe CS compared to pathogen treatment. Conspicuous DRR symptoms were observed in the roots from severe CS treatments compared to pathogen treatment (Supplementary Figs S10 and S11, Supplementary Video S1). Foliar biomass was also profoundly decreased in severe CS compared to control, severe DS and pathogen treatments in both the field location trials (Supplementary Figs S10 and S11, Supplementary Video S1). Additionally, higher incidence of Sclerotinia stem rot was observed in field location-3, and disease index for Sclerotinia stem rot was higher under severe CS compared to mild DS and mild CS (Supplementary Fig. S12). Also, high incidence of viral disease was observed in control and pathogen treatment plots in field location-2 (2016–17); however, incidence decreased in the drought and combined stress plots (Supplementary Fig. S13). Overall, we observed a high incidence of both BRR and DRR under severe CS, moderate CS and severe DS stress compared to the pathogen, control, mild DS and mild CS across both the locations throughout the years of study.

### Combined drought and pathogen stress decreased crop growth and yield

Specific leaf area (SLA) is an essential physiological trait representing the plant growth^24,25^. It represents the leaf biomass distribution compared to leaf area^25,26^. Also, SLA has been shown to have a positive correlation with rainfall^25^. We assessed the growth of the plant under different stress treatments by estimating SLA. Severe CS showed around two-fold decreased SLA compared to control and pathogen treatments in field location-1. Similarly, severe CS and severe DS showed around 1.2-fold reduction in SLA compared to control and pathogen in this field location-2 (Supplementary Fig. S14). Decreased SLA indicates the negative impact of combined stress and drought stress on plant growth. Photosynthetic parameters such as photosynthetic rate, stomatal conductance, and transpiration rate were also significantly reduced in severe DS and severe CS compared to control and pathogen stress in field location-1 (Supplementary Fig. S15A–C).

The yield in the field experiment was determined by estimating the total seed weight (gm/m^2^). Yield in severe CS and moderate CS was significantly decreased compared to pathogen stress and control (Table 3). Yield reduction in severe CS varied from 1.7 to 4.2-fold compared to pathogen treatment and 2.3 to 4.8-fold compared to control. On the contrary, severe CS did not show a significant difference in yield compared to severe DS probably due to the insignificant difference in DRR disease incidence and very less difference in BRR disease incidence between these two treatments. Moderate CS showed 1.4 to 2.2-fold reduced yield compared to the pathogen, and 1.8-fold to 2.3-fold compared to control. However, moderate CS too did not show a significant difference compared to moderate DS. The yield was also decreased in severe DS and moderate DS compared to control (Table 3). We encountered variations in yield data in some years which could probably be due to environmental variations during those periods (Supplementary File S2). Overall, the data indicate that drought has a significant impact on yield reduction in chickpea. Yield data also showed a high correlation between the field trials (*r*-value ranged from 0.82 at *p* < 0.05 to 0.97 at *p* < 0.001; Supplementary Table S4). It indicates that the yield trend among the treatments was well replicated in all field trials. Yield showed an inverse correlation with BRR incidence in all the field trials except for field location-2 in 2015–16 (*r*-value ranged from −0.81 at *p* < 0.05 to −0.94 at *p* < 0.001; Supplementary Table S5). Similarly, yield also exhibited inverse correlation with DRR incidence (*r*-value varied from −0.72 at *p* < 0.05 to −0.89 at *p* < 0.01; Supplementary Table S6).

**Table 3:** Impact of drought and combined stress on yield in two different field locations from the year 2015 to 2018.

Treatment	2015-16		2016-2017		2017-2018	
	Field-1	Field-2	Field-1	Field-2	Field-1	Field-2
Control	181.5^a^	176.8^a^	212.95^a^	176.59^a^	210.5^a^	182.18^a^
Mild DS	165.6^a^	172^ab^	135.4^b^	159.54^ab^	162.05^abc^	123.62^b^
Moderate DS	89.8^bc^	96.05^c^	98.68^bc^	155.43^ab^	115.5^bcd^	99.9^bcd^
Severe DS	60.6^c^	76.4^c^	73.25^cd^	132.49^bc^	88.56^cd^	72.51^d^
Pathogen	166.8^a^	136.5^abc^	188.25^a^	173.85^a^	182.16^ab^	171.44^a^
Mild CS	107.2^b^	114.1^bc^	86.1^c^	140.62^abc^	147.08^abc^	116.58^bc^
Moderate CS	85.2^bc^	92.5^c^	62.75^cd^	136.23^bc^	112.78^bcd^	76.08^cd^
Severe CS	50.4^c^	76.4^c^	44.15^d^	114.21^c^	58.82^d^	72.02^d^
**Grand mean**	**113.39**	**117.6**	**112.69**	**148.63**	**134.68**	**114.29**
**CV**	**16.28**	**29.24**	**14.29**	**13.92**	**26.12**	**24.81**
**LSD at** ***p*** ** < 0.05**	**43.652**	**60.214**	**38.085**	**36.244**	**83.185**	**41.69**

^*^Yield value represented in the table are for g/m^2^. Means of treatments are average of 2–4 block replicates. RCBD Two-way ANOVA was used for the comparison of mean and significance was assessed by the least significant difference (LSD at *p* < 0.05) post hoc test. Statistics are performed column-wise. Different letters (a, b, c, d) in a column denote significant difference between mean at *p* < 0.05. DS = drought stress; CS = combined stress; CV = coefficient of variance; LSD = least significant difference. Field location-1 = NIPGR, New Delhi; Field location-2 = GKVK, Bengaluru.

### Pot experiments confirmed the increase in DRR and BRR incidence under drought stress

**Figure 1:**
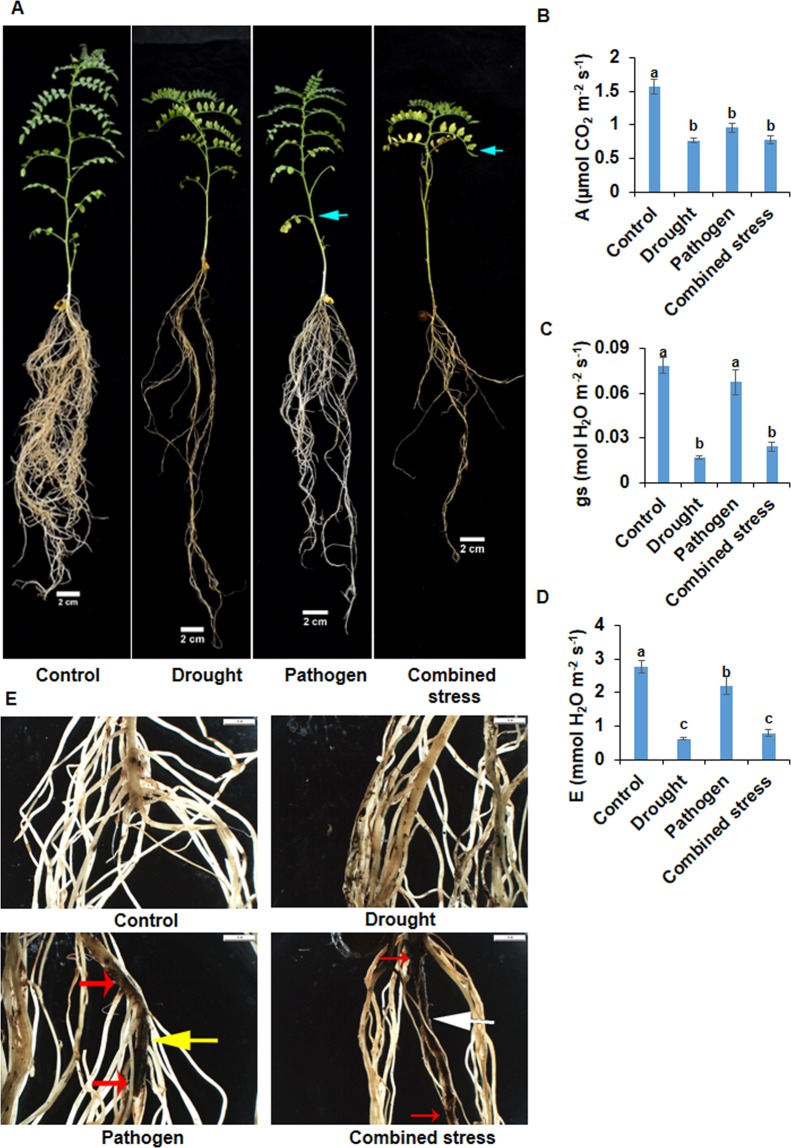
The morpho-physiological response of plants subjected to combined drought and *F. solani* infection. Chickpea genotype JG 62 was used to study the impact of combined drought and pathogen stress compared to drought only, and pathogen (*F. solani*) only stresses. Five days old nursery grown chickpea plants were used for drought, pathogen, combined stress treatments, and control. Chickpea root was immersed in *F. solani* spore suspension (1.1 × 10^5^ spores/ ml) for four hours to impose *F. solani* infection to pathogen only and combined stress treatments. For control and drought treatment, chickpea roots were dipped in sterile RO water for the same duration. Chickpea plants were re-planted into pots after four hours. Water withholding, to impose drought, was initiated five days after replanting into pots. Drought level (FC-35%) was achieved on 16th day after drought initiation and combined stress was counted from then. Disease symptoms at a morphological level were examined on fifth-day post combined stress treatment. Symptoms such as leaf yellowing (cyan arrow), root blackening, and root rot were observed in pathogen and combined stress treatment plants (**A**). Roots from control, drought, pathogen, and combined stress treatments were collected and observed under the 0.5 XPF objective of SZX16 Stereo Microscope (**E**). Scale bar represents 1 mm. Black root rot was evident in primary root (the distance between red arrows) of combined stress treatment. Additionally, pathogen treatment plants showed the emergence of lateral root (yellow arrow) and combined stress plants showed lack of lateral roots (white arrow). Leaf gas exchange parameters such as photosynthetic rate (**B**), stomatal conductance (**C**) and transpiration rate (**D**) were measured in the fourth leaf from the top in each treatment. Bar graph represents the average of three biological replicates ± SEM. Statistical significance was tested by two-way ANOVA followed by LSD All-Pairwise Comparisons post-hoc test. The different letters above each column represent significance difference between means at *p* < 0.05.

**Figure 2:**
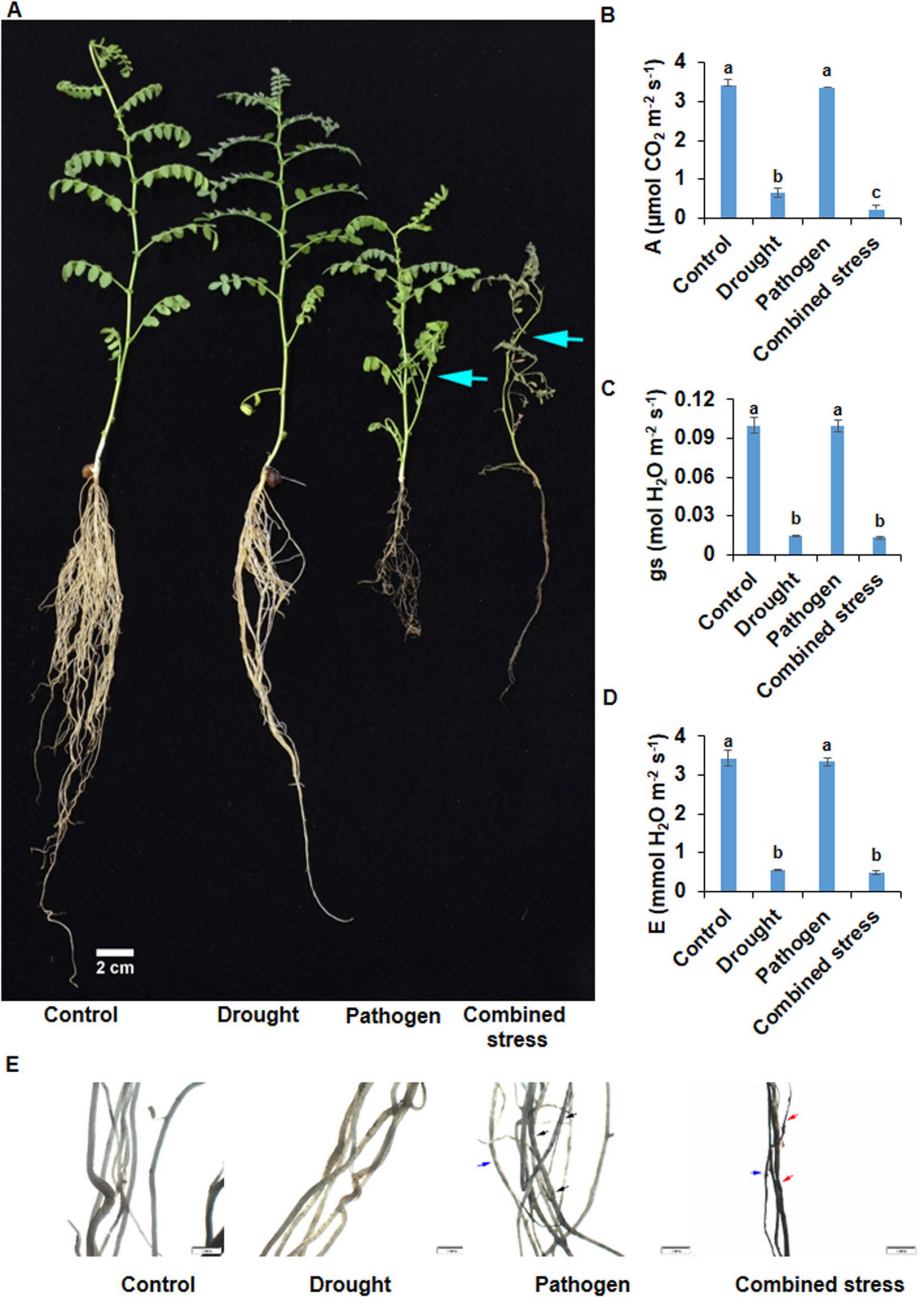
The morpho-physiological response of plants subjected to combined drought and *R. bataticola* infection. Drought, pathogen (*R. bataticola*) infection, and combined drought and pathogen infection were imposed on chickpea genotype JG 62. Chickpea plants for pathogen and combined stress treatments were grown in a sick pot containing *R. bataticola* inoculum. Plants for drought treatment and control were grown in autoclaved un-inoculated soilrite. Water with-holding for drought stress was initiated from fifth day post germination of chickpea. The desired drought (35% FC) level reached on the sixteenth day after water with-holding for both drought and combined stress treatments. Control and pathogen treatment plants were maintained at 90% FC for the entire experimental period. The drought was maintained at 35% FC for further five days, and disease symptoms at a morphological level were examined on the fifth-day. Symptoms such as leaf drying (cyan arrow), shedding of lateral roots, and root rot were observed in pathogen and combined stress-treated plants (**A**). Roots of control, drought, pathogen, and combined stress treatment plants were collected, and necrosis (black arrow), root rot (red arrow) and root diameter (blue arrow) was observed under the 0.5 XPF objective of SZX16 Stereo Microscope (**E**). Scale bar represents 1 mm. Leaf gas exchange parameters such as photosynthetic rate (**B**), stomatal conductance (**C**) and transpiration rate (**D**) were measured in forth leaf from the top for all the plants under four treatments. Bar graph represents the average of three biological replicates ± SEM. Statistical significance was tested by two-way ANOVA followed by LSD All-Pairwise Comparisons post-hoc test. The different letters above each column represent significance difference between means at *p* < 0.05.

A pot experiment was conducted to understand the impact of drought stress on BRR and DRR disease development. Control, pathogen (pathogen only), drought (drought only) and combined drought and pathogen treatments were considered for the study (Supplementary Figs S16, S17 and S18). Field capacity of 35% was considered as severe drought stress for this experiment based on the previous studies^13^. Chickpea genotype PUSA 372 did not show any disease development under pathogen only or combined stress treatments in pot experiment (Supplementary Fig. S19) as it is moderately resistant to pathogen infection. Therefore, pot experiments were performed using disease susceptible chickpea genotype JG62. Combined and drought stress treatments significantly decreased leaf RWC (analysis was done after 16 days of water withholding) compared to control. On the contrary, pathogen treatment did not show any reduction in RWC compared to control. A similar trend was observed in both combined drought and *F. solani* (Supplementary Fig. S20A), and combined drought and *R. bataticola* (Supplementary Fig. S20B) experiments. A significant reduction in leaf exchange parameters (Fig. 1B–D and Fig. 2B–D) and leaf RWC (Supplementary Fig. S20) at 35% FC of soil in this study confirmed that 35% FC acts as severe drought stress for chickpea.

**Figure 3:**
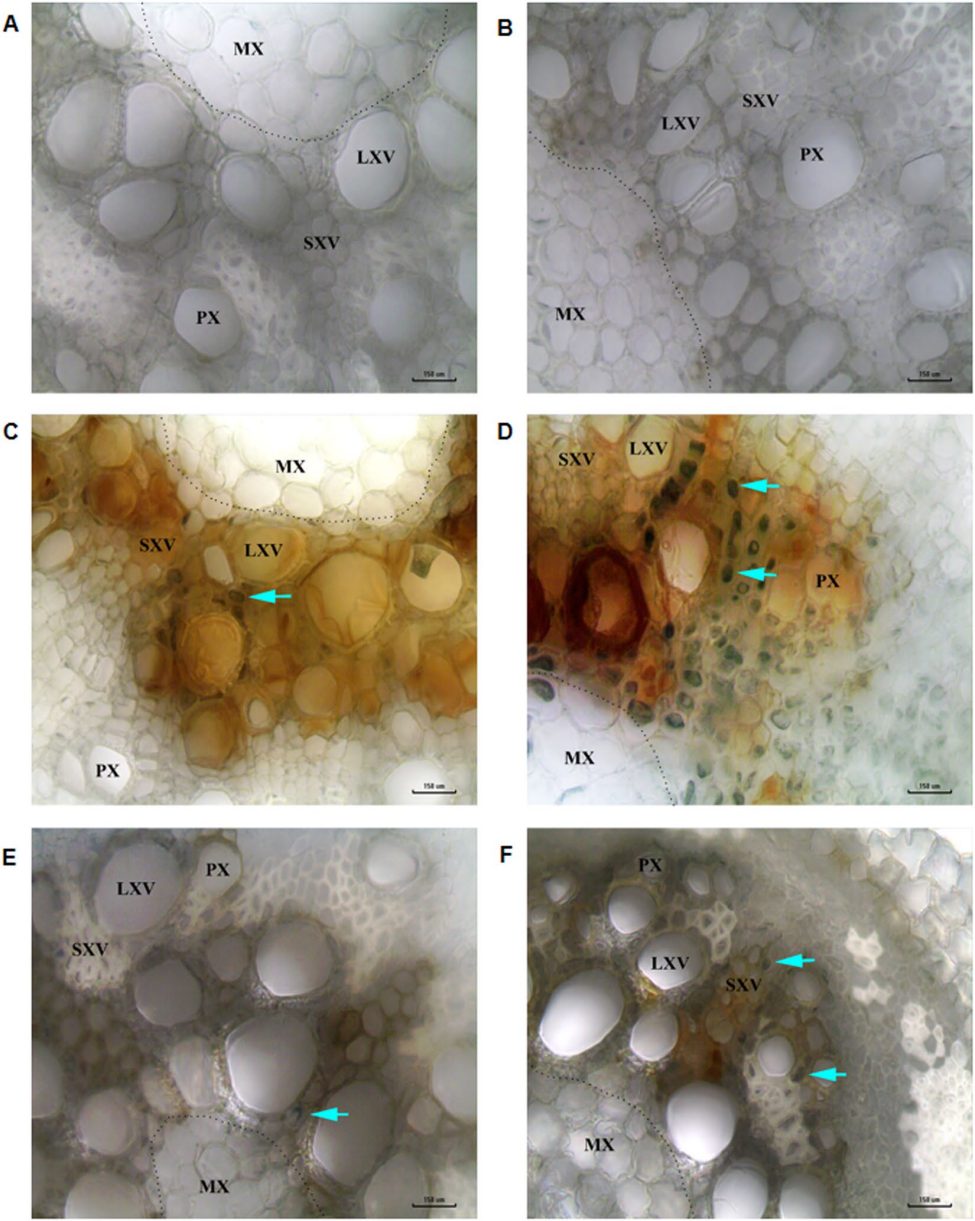
Bright field microscopy root images showing high fungal colonization in xylem regions of plants under combined stress. Transverse hand sections of plant roots from individual and combined stress treatments were stained with lactophenol aniline blue and observed under 40X objective with fixed 40X condenser of LMI BM-X microscope. No fungal mycelia were observed in the xylem of control (**A**) and drought (**B**) treated plants. Plant roots treated with *F. solani* infection have less number of stained mycelia (**C**) whereas plants treated with combined drought and *F. solani* infection showed large number of stained mycelia (**D**). Plant roots with *R. bataticola* infection (pathogen only) showed less stained mycelia (**E**) whereas plants treated with combined drought and *R. bataticola* showed large number of stained mycelia (**F**). The cyan arrow indicates the aniline blue stained fungal mycelia. Scale bar represents 150 µm, microscopy was done with three technical replicates. Two independent experiments showed similar result. MX, metaxylem; PX, protoxylem; LXV, large xylem vessel; SXV, small xylem vessel; dotted line demarcates the metaxylem and protoxylem. Note: the blue arrows are for pointing out stained fungal mycelia and not for quantification. Further, number of stained mycelial structures were counted under each microscopic field and the values are 20 ± 6 (**C**), 94 ± 5 (**D**),19 ± 2 (**E**) and 53 ± 5 (**F**). Besides blue staining, the dark brown colour is due to infection (see Supplementary Fig. 23). The difference in diameter of xylem vessels between control and drought could be due to treatment effect. All the images are captured under white balance background.

Chickpea plants under *F. solani* (pathogen), and combined drought and *F. solani* treatments, when analyzed for disease symptoms at the morphological level, showed necrotic lesions, blackening of primary root and reduction in lateral root number (Fig. 1A,E). Furthermore, a significant reduction in lateral root numbers and necrosis of root were observed in combined stress as compared to pathogen treated plants (Fig. 1A,E). Also, extended leaf yellowing of more than six leaves was observed in combined stress whereas pathogen only plants showed yellowing of only two lower leaves on five days post combined stress treatment (Fig. 1A, Supplementary Fig. S21). The disease severity index based on foliar symptoms was very high (45 DSI) for the combined stress compared to pathogen only (Supplementary Fig. S21). Also, fungal structures (cyan color, big arrow, stained with lactophenol aniline blue) was observed more (94 ± 5, fungal structures) in root transverse section (TS) of combined stress-treated plants (Fig. 3D) and less (20 ± 6, fungal structures) in the pathogen treated plants (Fig. 3C). All the disease parameters reflected severe BRR occurrence in combined stress compared to pathogen only treatment.

We examined the net impact of combined stress over individual stresses by assessing photosynthetic parameters and observed a two-fold decrease in photosynthetic rate in combined stress and drought stress treatments compared to control. Pathogen treatment also showed a 1.5-fold decrease in photosynthetic rate compared to control thus indicating the significant impact of BRR disease on photosynthesis (Fig. 1B). However, combined stress did not show a significant difference in photosynthetic rate compared to drought and pathogen treatments (Fig. 1B). Stomatal conductance was reduced by 3.2-fold in combined stress and 4.7-fold in drought stress compared to control. Combined stress showed a 2.8-fold reduction in stomatal conductance compared to pathogen stress, but no significant difference compared to drought only stress (Fig. 1C). Similarly, a significant reduction in transpiration rate was observed in combined stress compared to control (3.5-fold) and pathogen treatments (2.8-fold reduction); however, it did not vary compared to drought only stress (Fig. 1D). Drought (4.4-fold) and pathogen (1.2-fold) treatment also showed a decrease in transpiration rate compared to control (Fig. 1D).

We also assessed the disease severity of DRR under combined stress and compared it with individual drought, *R. bataticola* (pathogen only) and with control in a pot experiment. *R. bataticola* (pathogen only) and combined drought and *R. bataticola* treated plants showed root rot and reduction in lateral root number (Figs 2A and 2E,F). Moreover, combined stress-treated plants showed severe disease symptoms and severe reduction in lateral root number compared to pathogen treatment (Fig. 2A,E). Fungal mycelial like structures were observed in root TS of both combined stress and pathogen treated plants (Fig. 3E,F); however, the root of the combined stress plants had more number of fungal structures (blue stained structures) (Fig. 3F) compared to pathogen treatment (Fig. 3E). No disease symptoms developed in control and drought treatment (Figs 2A and 3A,B). The results confirmed that drought stress increased the severity of DRR in chickpea.

The photosynthetic rate in combined drought and *R. bataticola* stress was severely reduced compared to control (14.5-fold), drought (2.7-fold) and pathogen treatment (14.2-fold) (Fig. 2B). Similarly, stomatal conductance (Fig. 2C) and transpiration rate (Fig. 2D) was highly reduced in combined stress compared to control and pathogen stress but not in comparison to the drought stress. Drought stress also showed around five-fold decrease in photosynthetic rate compared to control (Fig. 2B). Similarly, stomatal conductance (Fig. 2C) and transpiration rate (Fig. 2D) was decreased by around six-fold in drought compared to control. Moreover, the stomatal conductance and transpiration rate did not vary between control and pathogen treatments (Fig. 2B,C). The results indicate an added impact of combined stress on photosynthetic rate but not on the transpiration and stomatal conductance over individual stress. The reduction in leaf exchange parameters in pot experiment showed a significant correlation with field experiment (*r-*value mostly ranged from 0.98 to 0.99, at *p* < 0.01, Supplementary Fig. S15D).

Overall, the lab experiment displayed increased DRR and BRR disease severity under combined stress, and combined stress had a net negative impact on the photosynthetic rate of chickpea.

## Discussion

Pathogen and drought are two very commonly occurring important stresses in chickpea fields. Therefore, it is essential to understand the actual interactions of these stresses and subsequent outcomes in the field. Previously, several studies have documented the role of drought stress in the modulation of pathogen infection and effect of combined stress^9–19,27–37^. In this study, we aimed to explore the impact of drought stress on the interaction of soil-borne pathogens with chickpea (not just one pathogen) under the natural condition and intended to screen the most influenced diseases under drought. We also aimed to study the overall impact of combined drought and pathogen stress on plant growth and yield. In order to address these questions, we performed systematic field experiments with moderately resistant chickpea genotype, PUSA 372 in sick plots. Selection of moderately resistant genotype was based on the hypothesis that the differences between treatments could be visible only when plants break the barrier of resistance under unfavorable condition. We assumed that the true impact of drought would be observable only if the resistant variety can become susceptible under drought. In this study, we observed a very low incidence of BRR and DRR under the non-stress condition. In contrast, drought stress enhanced the susceptibility of chickpea to these two diseases in the field (Tables 1 and 2). Drought negatively influenced the chickpea’s resistant towards *F. solani*, and *R. bataticola*.

In contrast, chickpea PUSA 372 did not show disease symptoms in the pot (Supplementary Fig. S19). When we conducted pot experiment for combined stress studies using root rot susceptible chickpea genotype JG62, we observed increased vulnerability of chickpea to BRR and DRR diseases under drought stress (Figs 1 and 2). Taken together, results from both resistant and susceptible variety as well as the field and pot experiments showed that drought stress increases the root rot infection in chickpea. In an earlier report, drought has been found to increase the production of *R. bataticola* microsclerotia in soil, and high soil moisture inhibited the microsclerotia production^38,39^. Increased root exudation under high temperature was also reported to increase the *R. bataticola* infection in chickpea^40^. These could be the probable reason for increased disease incidence under drought in our study. However, the exact mechanism behind the increased incidence of the diseases under drought remains elusive.

Plants under combined drought and *F. solani* infection in our study had short and rotten primary root as compared to plants under pathogen only treatment (Fig. 1A). Similarly, combined drought and *R. bataticola* infected plants had short primary root as compared to pathogen only infected plants (Fig. 2A). In general, increase in root length density (RLD) and root diameter has been associated with drought tolerance trait in crops as root grows deep into the soil to acquire water^41–45^ and similarly, decreased RLD indicates drought susceptibility^46^. RLD is highly correlated with plant growth and performance^46^. In our study, we observed a decrease in the RLD and root biomass under severe combined stress indicating that the root rots have compromised the ability of plant performance under drought. However, severe drought also showed less root biomass compared to control treatment, and this could be due to drought susceptibility of PUSA 372. Similarly, root system architecture plays a role in deciding susceptibility or resistance of plant for pathogen infection. Higginbotham *et al*. (2004) reported that increased root length decreases the infection of *Pythium debaryanum* and *Pythium ultimum* (causal agents of root rot) in *Triticum aestivum*^47^. Therefore, we assume that low RLD could be one of the reasons for increased disease susceptibility under drought and combined stress in our study.

In this study, we observed a reduction in photosynthetic rate, stomatal conductance and transpiration rate under both combined drought and *F. solani*, and combined drought and *R. bataticola* infection as well as under drought stress only, *F. solani* only stress in both pot and field experiments (Figs 1B–D and 2B–D, Supplementary Fig. S15). Earlier, Burman and Lodha (1996) and Mayek-Pérez and group, (2002) also reported a decrease in leaf turgidity, shoot water potential, and transpiration in *Vigna unguiculata*^48^ and *Phaseolus vulgaris*^49^ under combined drought and *Macrophomina phaseolina* infection. The rate of transpiration and stomatal conductance has an important role in maintaining cooler canopy. Reduction in transpiration causes increased canopy temperature under drought stress^50,51^. Under combined stress, root rot enhances the drought effect by inhibiting the water uptake due to damaged root, and thus, reduces leaf water potential and gas exchange. In our study, we found increased canopy temperature under combined stress and severe drought treatments (Supplementary Fig. S8C,D). Earlier, various other combined drought and pathogen stress studies have also reported increased canopy temperature under combined stress. For example, increased canopy temperature was noticed in *Beta vulgaris* (sugar beet) under combined drought and *Pythium aphanidermatum* (causal agent of root rot) infection. Similarly, in *Gossypium spp*. canopy temperature increased under drought and *Phymatotrichum omnivorum* infection (causal agent of Phymatotrichum root rot)^52^. *P. vulgaris* also displayed high canopy temperature and reduced stomatal conductance under combined drought and *M. phaseolina* (causal agent of charcoal rot) infection^49^. Combined stress also affected the yield and specific leaf area. We observed a reduction in yield and SLA under severe combined stress and drought stress in the field experiment (Table 3 and Supplementary Fig. S14). Pang *et al*. showed that stomatal conductance, transpiration rate, cumulative filled pod number, and cumulative seed number decreases even with the 40% reduction in the available soil moisture^45,46^. Moreover, a 60% reduction in soil water decreases leaf water potential and the photosynthetic rate. Further, 80% decrease in soil moisture affects the maturation of pods, and they claimed increased ABA as a cause for the reduction in seed setting^45,46^. Yield in our study also showed an inverse relation with drought level (Table 3).

The data we generated for plant-pathogen interaction under drought and the overall impact of combined stress on yield is from one of the few systematic studies available in literature till date for the combined stress prediction. Such studies are needed for simulation modeling for the prediction of disease incidence under drought stress and to understand the consequence of combined stress for future years and new field locations. Simulation models have been used to predict the climate change impact for the larger areas, different soil types, climatic regions, and crops from limited experimental data using various physiology-based crop simulation methods^23^. Previously, simulation models have also been employed for disease prediction under changing climatic scenario^53,54^. In another instance, Garcia *et al*. (2008) developed an agro-meteorological disease model to predict the sowing dates with the least risk for the potato late blight disease infection^55^. We, in this direction, attempted a case study to predict chickpea seed yield under drought stress based on the parameter similar to the field experiment using the DSSAT (Decision Support System for Agrotechnology Transfer) simulation-modeling tool. We used weather data, soil parameters, irrigation schedule, fertilizer, and fungicide information from our field experiment and further compared the predicted yield with the actual yield for the same experiment (Supplementary Fig. S22). We found a similar trend from both field experiment and DSSAT prediction output for yield. Thus, we assume that a similar drought-pathogen module can be developed for the prediction of drought pathogen interaction and the severity or incidence of the disease under certain irrigation level.

In conclusion, in our field and pot experiments, we observed that combined drought and pathogen infection had a significant negative impact on chickpea performance and yield as compared to individual treatments. However, a significant difference between the effect of moderate and severe combined stress was not observed. In the pot experiment, the root rot and drought under combined stress was found to be the reasons behind the decrease in photosynthetic rate, transpiration, and stomatal conductance and reduced plant growth. Similar observations were made in the field study. Understanding the drought impact on pathogen interaction in chickpea at multiple locations by field trial and simulation modeling is needed.

## Material and Methods

### Field experiment

#### Plant material and field location

*Cicer arietinum* var. PUSA 372 seeds (chickpea, from Indian Agricultural Research Institute, Pusa, New Delhi) were used in the study. It has a lifespan of 140–145 days with an average yield of 2.5–3.0 t/ha. It is moderately resistant to wilt, blight and root rot (Directorate of Pulses Development, http://dpd.gov.in/). The field experiment was conducted at three different locations in India. The field location-1 was located at National Institute of Plant Genome Research (NIPGR), New Delhi (28.6139°N, 77.2090°E) and the field location-2 was situated at Gandhi Krishi Vigyan Kendra (GKVK), University of Agriculture Sciences (UAS) Bangalore (12.9716°N, 77.5946°E), Karnataka. The experiment in field location-1 and field location-2 was conducted for a period of four years from 2014 to 2018. The field location-3 was located at Agricultural Research Station & Krishi Vigyan Kendra (KVK) field station, Tamil Nadu Agricultural University, Virinjipuram, Tamil Nadu (12.9165° N, 79.1325° E) and the experiment here was conducted for one year (2014–15). Each year, the experiment was performed during the chickpea cultivation period, i.e., from October to March. Seeds coated with and without fungicides were sown in the field with 10 × 30 cm spacing between plants and rows respectively in drought only and combined stress treatments. DAP (Diammonium phosphate, Goel Fertilizer Company, Tulsipur), MOP (Muriate of Potash or Potassium chloride, Asha Agro Chemical, and Fertilizer, Dharwad) and Urea (Indian Agro Service, Ananda Nagar) fertilizers were applied in three phases, half as basal application and two quarter applications to fulfill the need of nitrogen (30 kg/h), phosphorus (60 kg/h), and potassium (25 kg/h). The field location-1 had an area of 1 × 1 m^2^ (two such separate plots) for each treatment plot with 30–40 plants in each. The field location-2 had a field area of 2 × 2 m^2^ (two such separate plots) with around 120–130 number of plants in each plot. Field location-1 contained loam type soil, and field location-2 contained sandy clay soil. Details of soil composition, characteristics, and daily weather data for all the years for each field are provided in Supplementary Table S1 and Supplementary File S2, respectively.

#### Treatments and stress imposition

The experimental design included seven different treatments (one pathogen only, three different levels of drought only stress and three combined stresses with different levels of drought stress) along with control in a randomized complete block design (RCBD) (Supplementary Figs S2 and S3). Three different levels of drought stress namely, mild drought stress (mild DS), moderate drought (moderate DS) and severe drought (severe DS) were imposed in the field treatments by regulating the number of irrigations. Mild drought treatment plots were irrigated once in every 15 days, moderate drought treatment plots were irrigated at an interval of 25 days, and severe drought treatment plots were irrigated once in every 30 days. However, the control plots and pathogen plots were irrigated every ten days. Similar drought stress levels were followed in combined stress treatments namely mild combined stress (mild CS), moderate combined stress (moderate CS) and severe combined (severe CS) treatment plots following the similar irrigation schedule (Supplementary Fig. S2). Fungicide seed treatment was undertaken in the control and drought stress treatments (Supplementary Fig. S2). For control and individual drought stress, seeds before sowing were treated with a mixture of Bavistin (50% WP Carbendazim, Hindustan Antibiotics Limited, Pune) and SAAF (Carbendazim 12% Mancozeb 63% WP, United Phosphorus Limited, Mumbai) in the concentration of 10 g/kg of seeds.

Further, Bavistin and SAAF fungicides were applied to the soil (2 kg/ha and 1 kg/ha, respectively) for three times. The experiment was conducted at three different locations namely field location-1, field location-2, and field location-3 as mentioned above. The field plots mentioned in this study have long-term ‘sick plots’ maintained for various disease studies. Overall, sick plots in these locations are known to harbor inoculums for the fungal, bacterial and viral pathogens of chickpea. The field location-1 had each treatment plot with 30–40 plants in each (Supplementary Fig. S4). The treatment plots in field location-1 were under rainout shelter. The field location-2 and field location-3 had around 120–130 number of plants in each plot (Supplementary Fig. S5). In each location, the treatments were replicated in four, and RCBD–based plot design was used (Supplementary Figs S3, S4 and S5).

#### Soil moisture content

Soil moisture content at field location-1 was measured every alternate day using Lutron PMS-714 soil moisture meter (Lutron Electronic Enterprise Co., Ltd., Taipei, Taiwan) at a depth of 15 cm from the surface for all the treatment plots. It was measured from a minimum of three different points for every treatment plots. Average of four block replicates were plotted for the comparison.

#### Canopy temperature

Field canopy temperature was measured using a Fluke Infrared Thermometer (Fluke Ti32, Fluke Corporation, Washington, USA). The thermal images (Supplementary Fig. S8E) were taken for each treatment plot at around 11 am, and the image was processed using SmartView 4.1 (Fluke Corporation, MN, USA). The temperature was recorded in oF for 30–35 random spots. Average of 30–35 spots were used for data analysis.

#### Specific leaf area

Leaves from 3–5 plants from each treatment plot were collected, and leaf area was measured using WinDIAS Leaf area meter (Delta-T- Devices Ltd, UK). Further, the leaves were oven dried and weighed. Specific leaf area (cm^2^/g) was calculated using the following formula

$$Specific\,leaf\,area\,(cm\,square/g)=\frac{area\,of\,leaf\,(cm\,square)}{dry\,weight\,of\,leaf\,(g)}$$

#### Disease identification and calculation of incidence and index

Various diseases mentioned in Supplementary Fig. S9 was recorded from experimental fields. Diseases were identified based on their typical symptomatic features. For instance, dried leaves, taproot devoid of lateral roots and finer roots and the presence of dark-colored microsclerotia on the inside as well as outside of the root were associated with DRR disease. Leaf yellowing, the presence of white fungal mycelia on the roots and black colored root were the symptoms associated with BRR disease. The whole plant wilting and brown discoloration of vascular tissue was associated with FW. Rotten collar region and root with white mycelia with macrosclerotia attached were the symptoms associated with CR^5,56^ (Supplementary Fig. S9). Plants showing DRR, BRR, FW and CR symptoms were collected from field and roots were surface sterilized using 2% sodium hypochlorite (Cat #1936071021, Merck life sciences, Mumbai, India) for two minutes and washed three to four times with autoclaved reverse osmosis (RO) water. Then, fungus from roots was cultured on potato dextrose agar media (Cat # 7109972, Becton, Dickson and company, USA), and fungus identity was initially confirmed by colony morphology as described in Summerell *et al*.^56^ and Nene *et al*.^5^ (Supplementary Fig. S9). Further, genomic DNA of *F. solani* and *R. bataticola* was isolated from fungal mycelia cultured on PDA media using DNAzol® Reagent (Cat # 10978021, Thermo Fisher Scientific, USA) and PCR with universal ITS primers [ITS1 (5′ TCCGTAGGTGAACCTGCGG3′) & ITS4 (5′TCCTCCGCTTATTGATATGC3′)] targeting internal transcribed spacer (ITS) was performed to confirm the fungus (Supplementary Fig. S9I,J)^57,58^. Furthermore, field isolated *R. bataticola* fungal culture was confirmed at Indian Type Culture Collection (ITCC, http://www.iari.res.in/index.php?option=com_content&view=article&id=1251&Itemid=1809), Indian Agricultural Research Institute (IARI), New Delhi based on mycelial morphology. Percent disease incidence (DI) for DRR, BRR, and virus was calculated in each plot for each disease types by the following formula

$$DI=\frac{number\,of\,infected\,plant\,in\,treatment\,plots}{total\,number\,of\,plants\,in\,treatment\,plots}\,\times \,100$$

#### Total grain yield

After the maturity at around 140 days, the plants in each treatment (about 30 plants) plots were harvested, and the pods were separated manually. The pods were sun-dried, and the seeds were extracted. Net yield for each treatment plot was calculated as grain weight in gm per m^2^ area. Average of four-block replicate was presented in Table 3.

### Pot experiments

#### Plant material and growth conditions

Chickpea genotype PUSA 372 and JG 62, procured from IARI, were used in the pot experiments. JG 62 is susceptible to DRR and BRR diseases^5^. Experimental chickpea seeds were surface sterilized with 2% sodium hypochlorite (Cat #1936071021, Merck life sciences, Mumbai, India) solution by vortexing for two minutes. Further, seeds were washed with autoclaved RO water five times for 30 seconds each. Soilrite (Keltech Energies Limited, Bangalore, India) was used as the soil medium in pot experiments. Plants were grown in a growth room with a temperature of 28 ± 2 °C and photoperiod of 16 h/8 h light/ dark and light intensity of 150 μmol m^−2^ s^−1^ and relative humidity of 52 ± 2%.

#### Combined drought and *F. solani* infection

Pot experiment comprised control, drought only, pathogen only (*F. solani*, pathogen) and combined drought and *F. solani* (combined stress) treatments (Supplementary Fig. S17). *F. solani* was procured from ITCC, IARI, New Delhi (*F. solani* ITCC 2751). Each treatment in the pot experiment included ten plants (ten biological replicates) at a time. Soilrite used in the experiment was autoclaved twice, dried under shade and 70 g was filled in each pot. Five days old chickpea plants from a nursery were infected by dipping the roots in *F. solani* suspension (1.1 × 10^5^ spores per ml) for four hours for pathogen and combined stress treatments. Roots of control and drought treatment were dipped in sterilized RO water for the same duration. Plants were re-planted after four hours into the pots containing soilrite. For drought and combined stress, drought imposition was initiated five days after the re-planting. Control and pathogen only treatments were well- watered to maintain 90% FC. Drought stress (35% FC, Ψ_w_ −1.0 MPa) was achieved in sixteen days, and it was maintained at 35% FC for the next five days. Outline of the protocol is provided in Supplementary Fig. S17.

#### Combined drought and *R. bataticola* infection

Pot experiment to study combined drought and *R. bataticola* also comprised control, drought only, pathogen only (*R. bataticola*, pathogen) and combined drought and *R. bataticola* (combined stress) treatments with ten biological replicates (in a single experiment, Supplementary Fig. S18). *R*. *bataticola* isolated from the field experiment was used for the pot experiments. Sterile soilrite was used for control and drought only treatments. The sick pot containing *R. bataticola* inoculum was used for pathogen and combined stress treatments. The sick pot was raised by inoculating five agar plugs (5 mm in diameter) of ten days old *R. bataticola* culture into overnight soaked and autoclaved chickpea seeds in a polythene bag. The inoculated seeds were incubated at 28 °C for 15 days. Later, the seeds with microsclerotia were mixed into dry soilrite in 50:100 (weight/weight) ratio. Surface sterilized seeds were sown at 2-cm depth in all treatment pots and bottom irrigated with water. Five days after germination, drought imposition was initiated by water withholding for drought only and combined stress treatments. Control and pathogen only treatments were well-watered (90% FC). Drought stress (35% FC) was achieved in 16 days, and it was maintained for the next five days at 35% FC. Outline of the protocol was provided in Supplementary Fig. S18.

#### Microscopic observations

Root region of plants subjected to individual and combined stress treatments was collected, washed thoroughly using the autoclaved RO water and fixed in 10% neutral buffered formalin (NBF) for 24 hours. NBF was prepared by dissolving 4.0 g of sodium dihydrogen phosphate, monohydrate (Cat # 7558807, Bio basic, Toronto, Canada) and 6.5 g of disodium hydrogen phosphate, anhydrous (cat # 7558794, Bio basic, Toronto, Canada) in 730 ml milli-Q water and 270 ml of 37% formaldehyde (Cat # 0493, Amresco, Ohio, USA). Then, hand sections of root were stained with lactophenol aniline blue for ten minutes followed by destaining for 20 minutes (twice) with chloral hydrate (2.5 g/ml) (GEC laboratories, Mumbai, India). The composition of lactophenol aniline blue is 20 ml lactic acid (Cat # 13025, Fisher scientific, Navi Mumbai, India), 20 ml phenol (Cat # 4557, Sigma, USA), 40 ml glycerol (Cat # 56815, Fisher Scientific, Mumbai, India) and 20 ml of milli-Q and 50 mg of aniline blue (Cat # GRM901, Himedia, USA). Then, sections were mounted with 40% glycerol on a slide and observed under 40X objective of LMI BM-X microscope (LMI microscope, England, UK). For the pictures on whole roots, images on disease symptom severity were taken under the 0.5 XPF objective of SZX16 Stereo Microscope (Olympus Corporation, Tokyo, Japan) (Supplementary Fig. S23).

#### Disease scoring and disease index

Disease severity index for BRR under pathogen only and combined stress treatments was calculated based on a foliar symptom of treatment plants at fifth-day post-combined stress treatment. Disease symptoms were assigned five scores from 0–4 based on foliar symptoms in the plant. Score 0 = all green leaves per plant, score 1 = two yellow leaves per plant, score 2 = four yellow leaves per plant, score 3 = six yellow leaves per plant, score 4 = eight yellow leaves per plant (Supplementary Fig. S21). Disease severity index (DSI) was calculated from ten biological replicates using the following formula^59^.

$$DSI( \% )=\frac{{\sum }_{0}^{n}(class\,frequency\times score\,of\,rating\,class)}{(total\,number\,of\,observation)\times (maximal\,disease\,index)}\times 100$$

#### Relative water content (RWC)

RWC in leaflets collected from pot experiment was determined using the protocol described earlier by Sinha *et al*. (2016)^13^. Fresh weight (FW) of the leaflet was recorded instantaneously after its collection and leaflets were hydrated by floating on de-ionized water for five hours at 22 °C until its full turgidity. Then, turgid weight (TW) was noted. Leaflets were then dried in an oven at 60 °C for two days until they reach the constant weight and then dry weight (DW) was measured. RWC was calculated using the following formula:

$${\rm{RWC}}\,( \% )=[\frac{{\rm{FW}}-{\rm{DW}}}{{\rm{TW}}-{\rm{DW}}}]\times 100$$

#### Measurement of gas exchange parameters

Leaf gas exchange was measured from three leaflets of the fourth leaf from the top using a LICOR-6400 XT (LI-COR, Lincoln, NE, USA). The photosynthetic rate (A, µmol CO_2_ m^−2^ s^−1^), transpiration rate (E, mmol H_2_O m^−2^ s^−1^) and stomatal conductance to H_2_O (gs, mol H_2_O m^−2^ s^−1^) were measured under irradiation of 200 μmol m^−2^ s^−1^, the CO_2_ concentration of 300 μmol mol^−1^ and block temperature of 28 °C. Readings were taken from three biological replicates. Leaf gas exchange for field experiment was measured using default factory chamber using natural photosynthetically active radiation (PAR). The measurements were taken from three random plants in each plot.

#### Statistical analysis

Since the field experiments included RCBD method, an average of block replicates of each treatment for field parameters were compared using RCBD two-way ANOVA with Fisher’s Least Significant Difference (LSD) post-hoc test or Tukey’s post-hoc test. Owing to the large variation within block replicates of treatments, BRR DI, DRR DI, soil moisture, canopy temperature, gas exchange parameters, and yield parameters were compared using Fisher’s Least Significant Difference (LSD) post-hoc test. Yield and disease incidence data were tested for the correlation using the Pearson correlation using GraphPad Prism 7 (GraphPad Software, CA, USA). The gas exchange data for pot experiments were presented as the average of three biological replicates and means were compared using RCBD two-way ANOVA followed by Fisher’s LSD. RWC data means were compared using one-way ANOVA and Tukey’s post-hoc test. RCBD two-way ANOVA analyses were done using Statistix10 software (Analytical Software, Tallahassee). One-way ANOVA was done using GraphPad Prism 7 (GraphPad Software, CA, USA).

## Supplementary information

Supplementary Figs. 1-23 and Supplementary Tables 1-6

Supplementary file 1-3

Supplementary File S4

Supplementary Video S1
