# Supplementary material for: Impact of drought stress on simultaneously occurring pathogen infection in field-grown chickpea
Source: Sci Rep. 2019 Apr 3;9:5577. doi: 10.1038/s41598-019-41463-z (PMC6447570; doi:10.1038/s41598-019-41463-z)
Supplement: Supplementary file 1 — Supplementary Figs. 1-23 and Supplementary Tables 1-6 [file 41598_2019_41463_MOESM1_ESM.pdf]

Impact of drought stress on simultaneously occurring pathogen infection in field-grown chickpea

Ranjita Sinha[1, #], Vadivelmurugan Irulappan[1, #], Basavaiah Mohan Raju[2], Angappan Suganthi[3, 4] and Muthappa Senthil-Kumar[1, *]

[1]National Institute of Plant Genome Research, JNU Campus, Aruna Asaf Ali Marg, New Delhi-110067 India

[2]Department of Crop Physiology, University of Agricultural Sciences, GKVK, Bangalore- 560065 India

[3]Agricultural research station and Krishi Vigyan Kendra, Tamil Nadu Agricultural University, Virinjipuram, Vellore - 632104 India

*corresponding author: skmuthappa@nipgr.ac.in,

[#]equal contribution

[4]Present address: Tamil Nadu Agricultural University, Coimbatore, 641003 India

Supplementary Figs. 1-23 and Supplementary Tables1-6

No. of pages: 40

doi.org/10.1038/s41598-019-41463-z

Supplementary Fig. S1

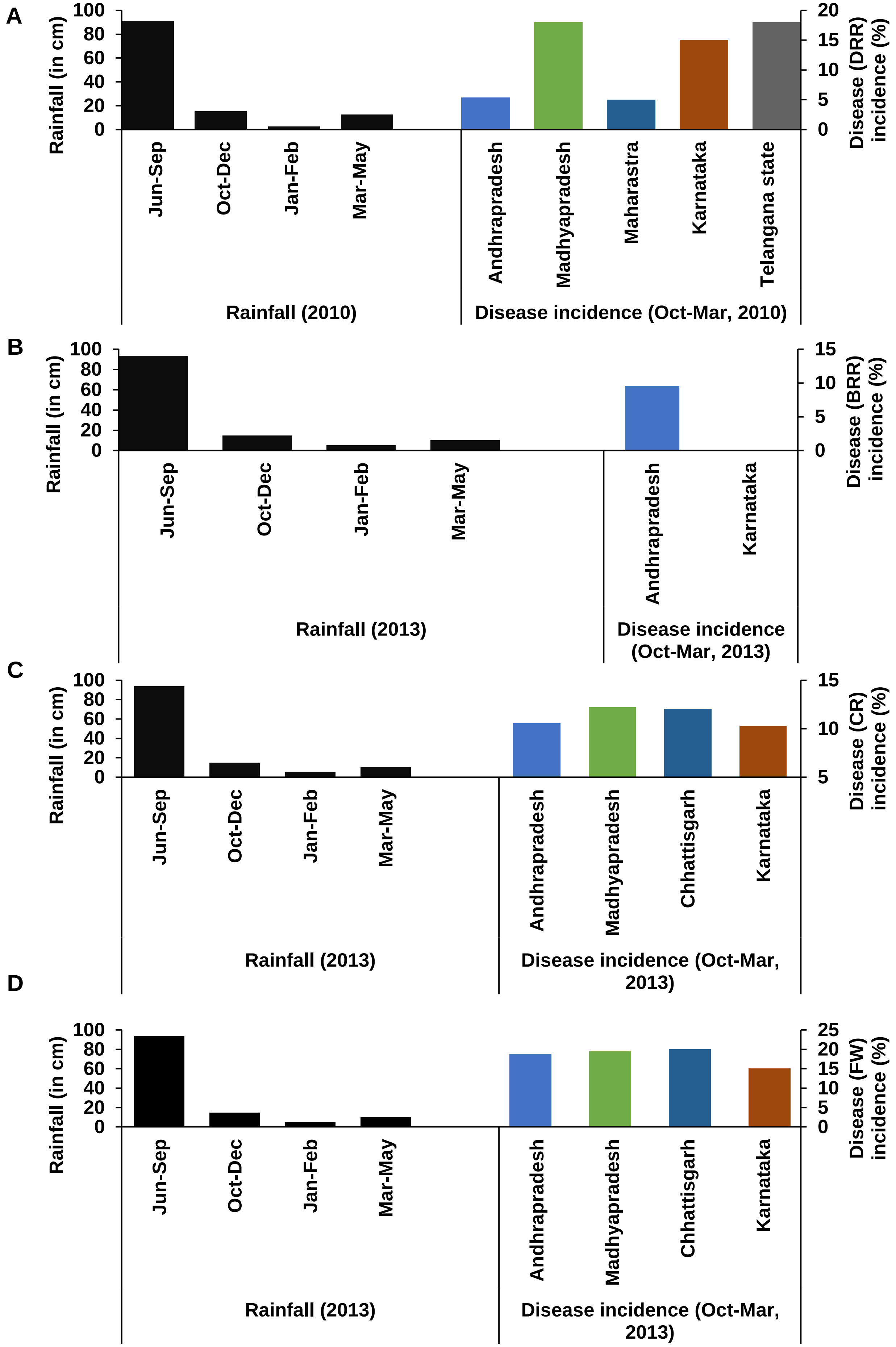

Supplementary Fig. S1. Simultaneous occurrence of drought stress and various chickpea disease across states in India. Rainfall data were downloaded for the year of 2010 and 2013 from https://data.gov.in and for the same years, data for the disease incidence was collected from the literure[1, 2 & 3] (**Supplementary File. S3**). Graph represents the co-occurrence of drought and economically important chickpea disease (Dry root rot (DRR) (A), Black root rot (BRR) (B), Collar rot (CR) (C) and Fusarium wilt (FW) (D)) in chickpea growing states (Andhra Pradesh, Telangana state, Madhya Pradesh, Maharashtra, Karnataka and Chhattisgarh) in India. The primary y-axis shows rainfall measured by rainguage in mm (converted into cm) and averaged for the whole country. The secondary y-axis shows the percentage of disease incidence.

Reference:

1. Ghosh, R., Sharma, M., Telangre, R., & Pande, S. (2013). Occurrence and distribution of chickpea diseases in central and southern parts of India. *American Journal of Plant Sciences*, *4*(4), 940-944.
2. Srinivas, P. (2016). Studies on dry root rot [*Rhizoctonia bataticola* (Taub.) Butler] of Chickpea (*Cicer arietinum* L.) (Doctoral dissertation, Professor Jayashankar Telangana State Agricultural University).
3. Wagh, P. (2015). Studies on dry root rot (*Rhizoctonia bataticola* taub (butler)) of chickpea (*Cicer arietinum*) (Doctoral dissertation, Indira Gandhi Krishi Vishwavidyalaya, Raipur).

Supplementary Fig. S2

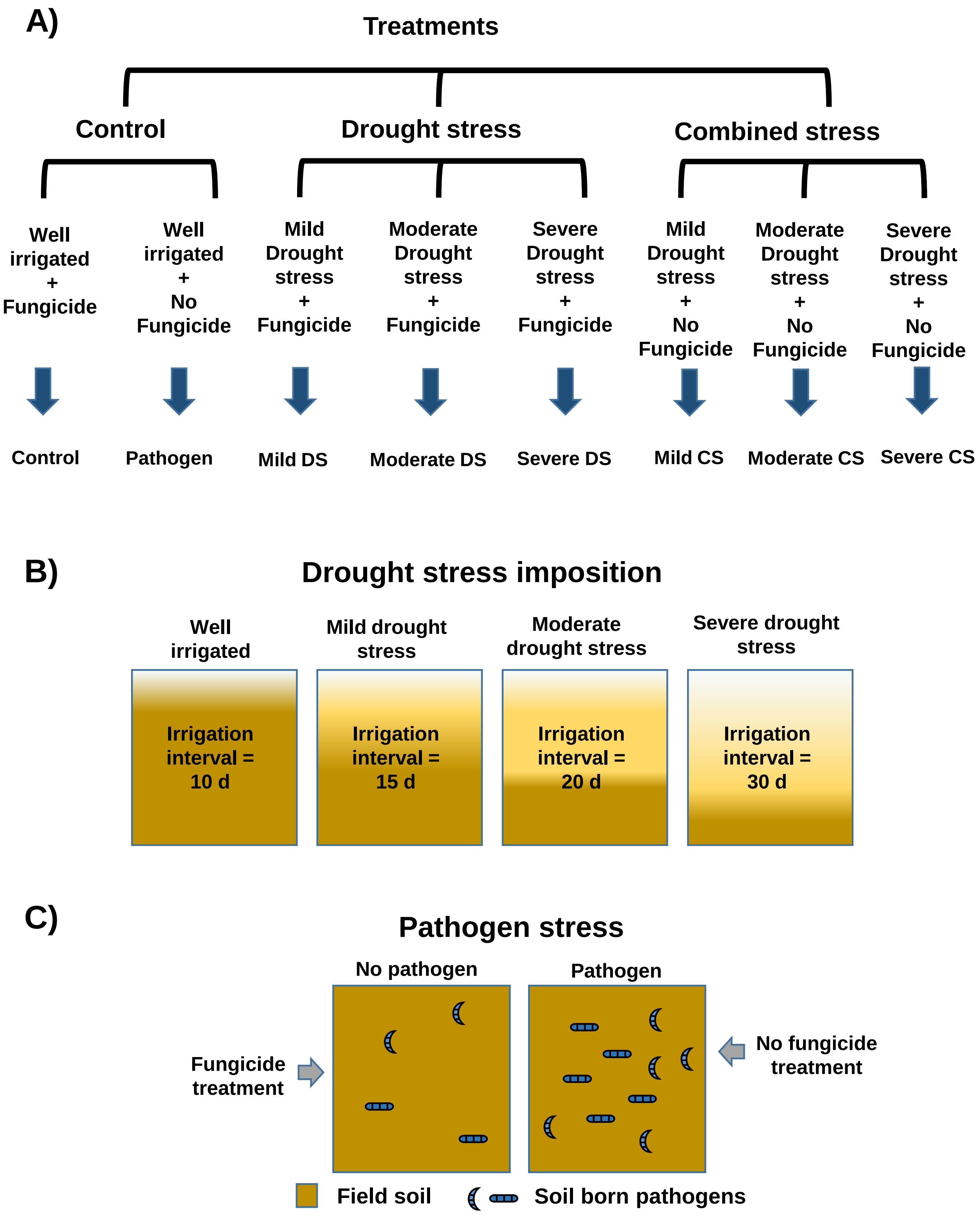

Supplementary Fig. S2. Illustration of individual and combined stress imposition method used in field experiments. A field experiment was conducted in a sick plot with chickpea var. PUSA 372. Altogether, this field experiment involved seven treatments namely, mild drought stress (mild DS), moderate drought stress (moderate DS), severe drought stress (severe DS), pathogen, mild combined stress (mild CS), moderate combined stress (moderate CS) and severe combined stress (severe CS) and control. Chart (A) represents all the treatments employed in the current study. DS and CS indicate drought stress and combined stress respectively. Figure (B) and (C) represent drought stress and pathogen stress imposition method respectively. Drought stress was imposed by decreasing the number of irrigations in the crop life cycle. Control and pathogen treatment plots were irrigated every ten days, mild DS and mild CS plots were irrigated once in every 15 days, moderate DS and moderate CS treatment plots were irrigated with a gap of 20 days, and severe DS and severe CS treatment plots were irrigated after every 30 days interval (b). Sick plot allows the natural incidence of pathogen in combined stress treatments however, fungicide Bavistin 50DF (Carbendazim 50% DF) in a concentration of 2 kg/ha and Mancozeb in a concentration of 1 kg/ha were used trice with intermittent application of SAAF (Carbendazim 12% + Mancozeb 63% WP, 1 kg/ha) during entire field experiments to control pathogen growth in control and DS treatment plots. Also, seeds were pretreated with Bavistin and SAAF for fungal control. Best agronomic practices were adopted for the crop growth in two field locations, field location 1 and field location 2, in India. It included application of DAP (Diammonium phosphate), MOP (Muriate of Potash or Potassium chloride) and Urea fertilizers in three phases, half as basal application and two quarter applications to fulfill the need of Nitrogen (30 kg/h), Phosphorus (60 kg/h), Potassium (25 kg/h). Also, Chlorpyriphos 50% EC pesticide (Nagraj 505) were applied to eliminate the incidence of insects and viruses via aphids. Weather conditions of field locations are provided in supplementary file S2, and details of the soil are provided in supplementary table S1.

Supplementary Fig. S3

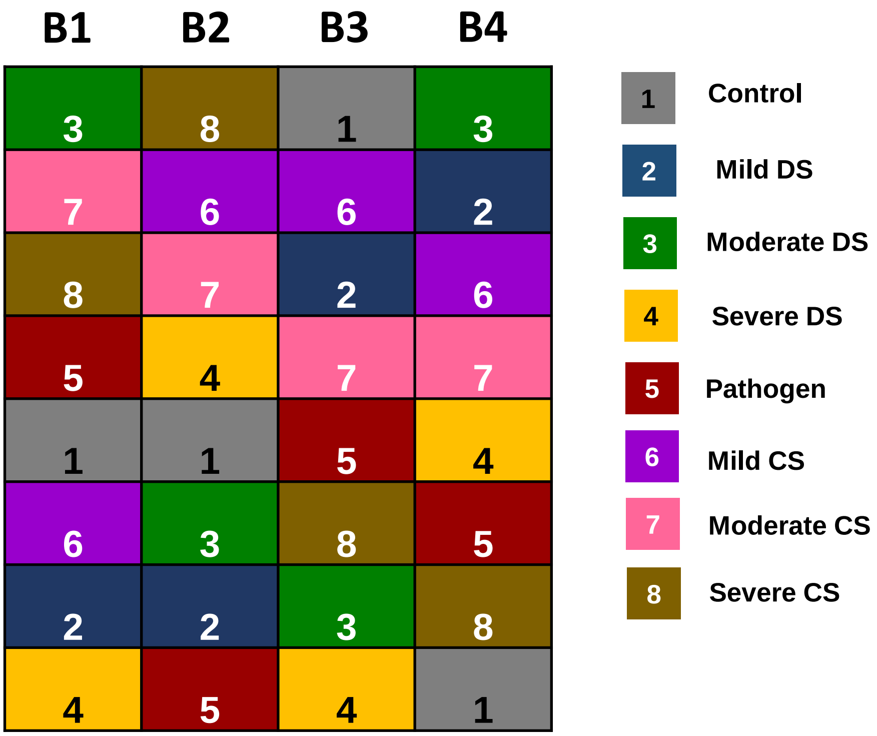

Supplementary Fig. S3. The layout of treatment plots in randomized complete block design for field location-1 and field location-2. The treatments in this experiment were conducted in a randomized complete block design (RCBD). Four blocks (B1, B2, B3, B4) were used for four replications. Each block contained all the seven treatments (supplementary Fig. S2) and control in random order. DS and CS is an abbreviation for drought stress and combined stress respectively. The Field location 1 had an area of 1x1 m^2 for each treatment plot with approximately 30 chickpea plants in each. The Field location 2 had a field area of 2x2 m^2 with around 120 number of plants in each treatment plots. Chickpea seeds were sown with 10 cm spacing between the plants and 30 cm spacing between the rows. Weather conditions of field locations are provided in supplementary file S2, and details of the soil are provided in supplementary table S1.

Supplementary Fig. S4

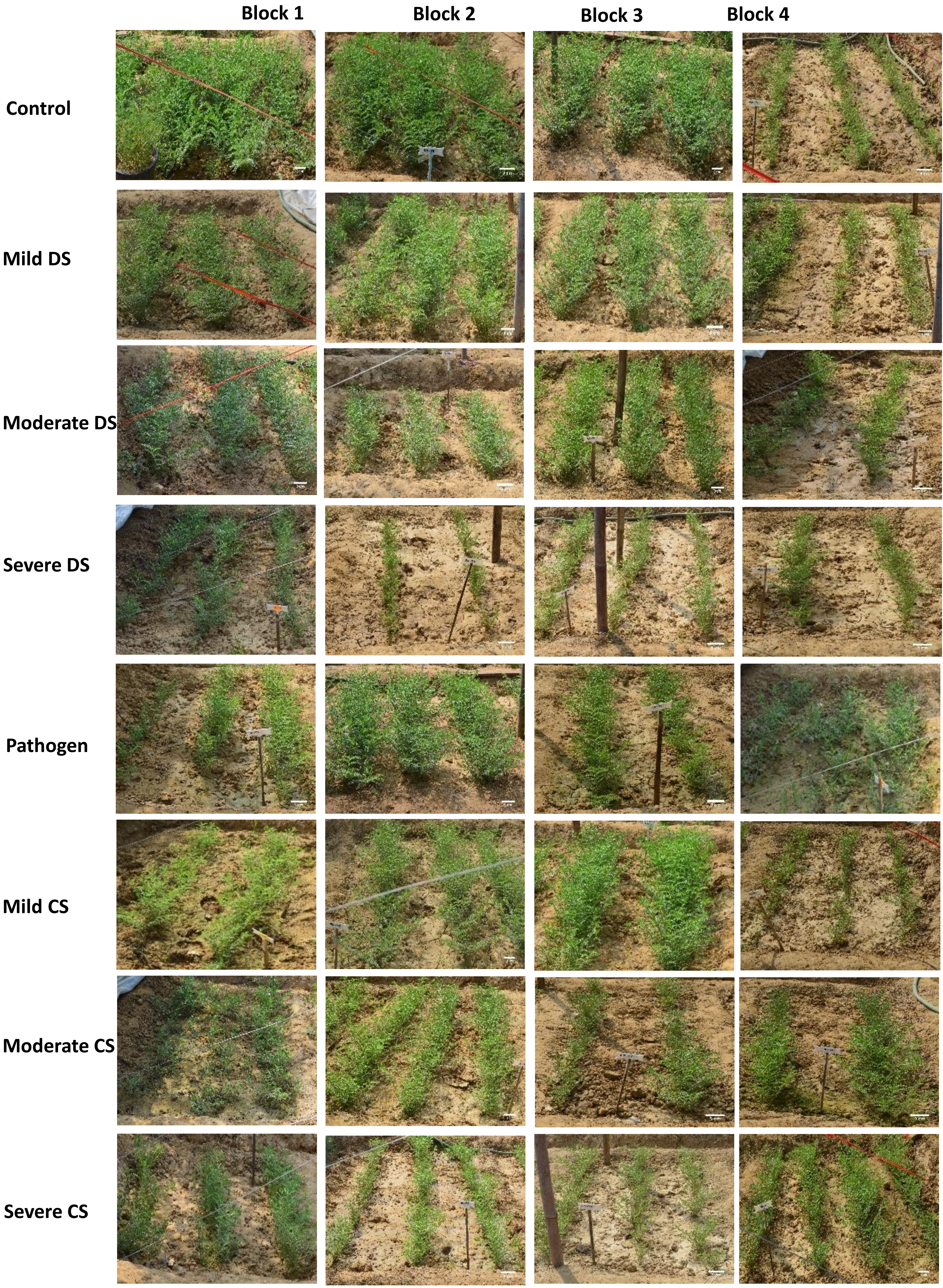

Supplementary Fig. S4: Plant growth in field location 1 experimental plot. Pictures are representing chickpea var. PUSA 372 growth in each treatment plots. Treatments (supplementary fig. S2) were randomized following RCBD design. Field design contained four blocks which represent four replicates, and each block contains all seven treatments and control. Each treatment plots in the figure contain 2-3 rows with around 30-40 plants in an area of around 1x1 m^2. The plants in the figure are eight weeks old with one month of drought stress in severe DS and severe CS. DS and CS represent drought stress and combined stress respectively. Plots in the figure are representation of field location-1 view showing different treatments. Pictures are not scalable and not for the final inference. Weather condition of growth is provided in supplementary file S2. Details of the soil type and characteristics are provided in supplementary table S1.

Supplementary Fig. S5

Block 1 | Block 2 | Block 3 | Block 4

Control

Mild DS

Moderate DS

Severe DS

Pathogen

Mild CS

Moderate CS

Severe CS

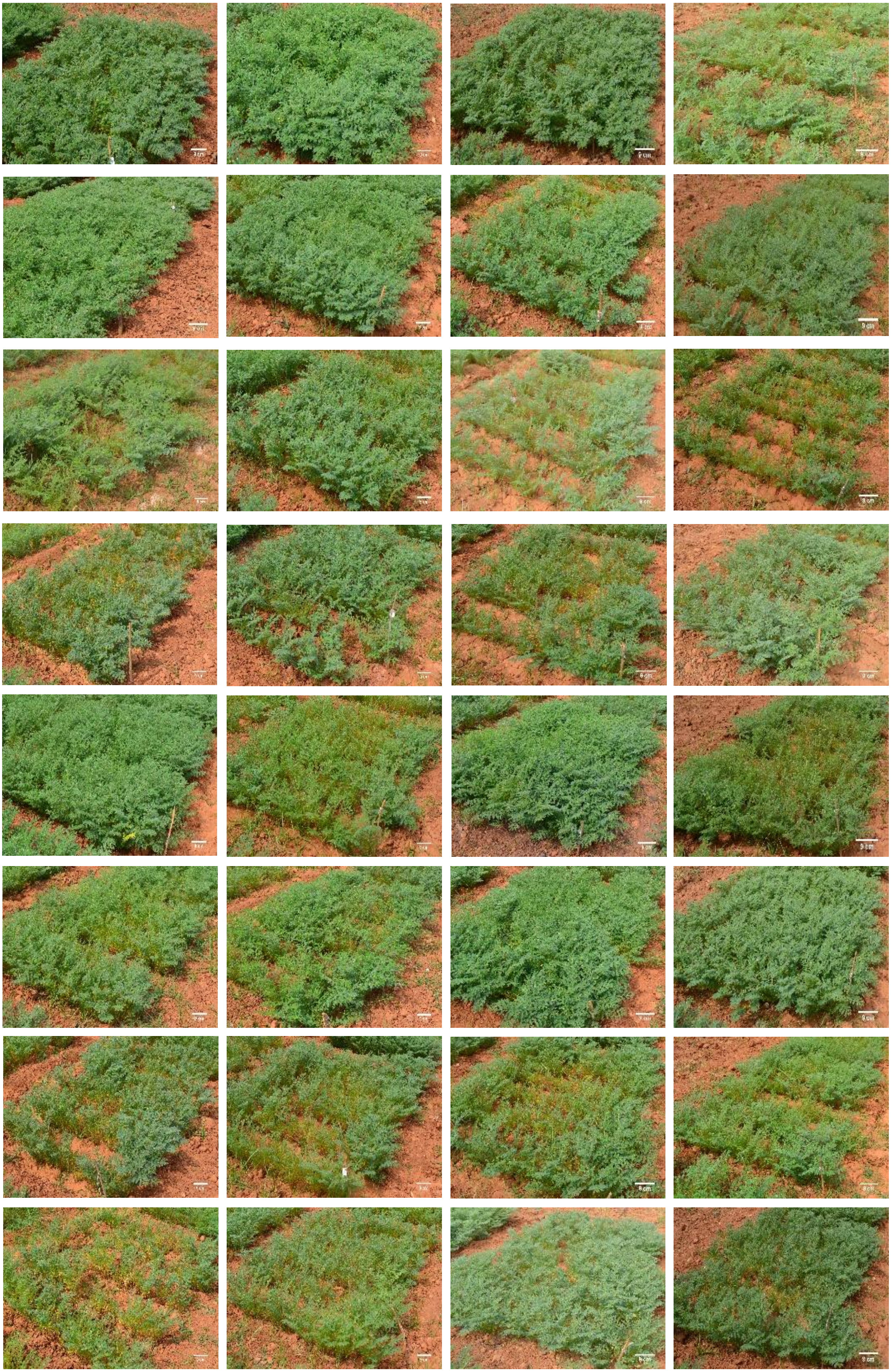

Supplementary Fig. S5. Plant growth field location-2 experimental plot. Pictures are representing growth of the chickpea plants in each treatments plots. Treatments (supplementary fig. S2) were randomized following RCBD design. Field design contained four blocks which represent four replicates, and each block contains all seven treatments and control. Each treatment plots has $4m^2$ of the area with around 120-130 plants. The plants in the figure are eight weeks old with one month of drought stress in severe DS and severe CS. Chickpea seeds were sown with 10 cm spacing between the plants and 30 cm spacing between the rows. DS and CS represent drought stress and combined stress respectively. Weather condition of growth is provided in supplementary file S2. Details of the soil type and characteristics are provided in supplementary table S1.

Supplementary Fig. S6

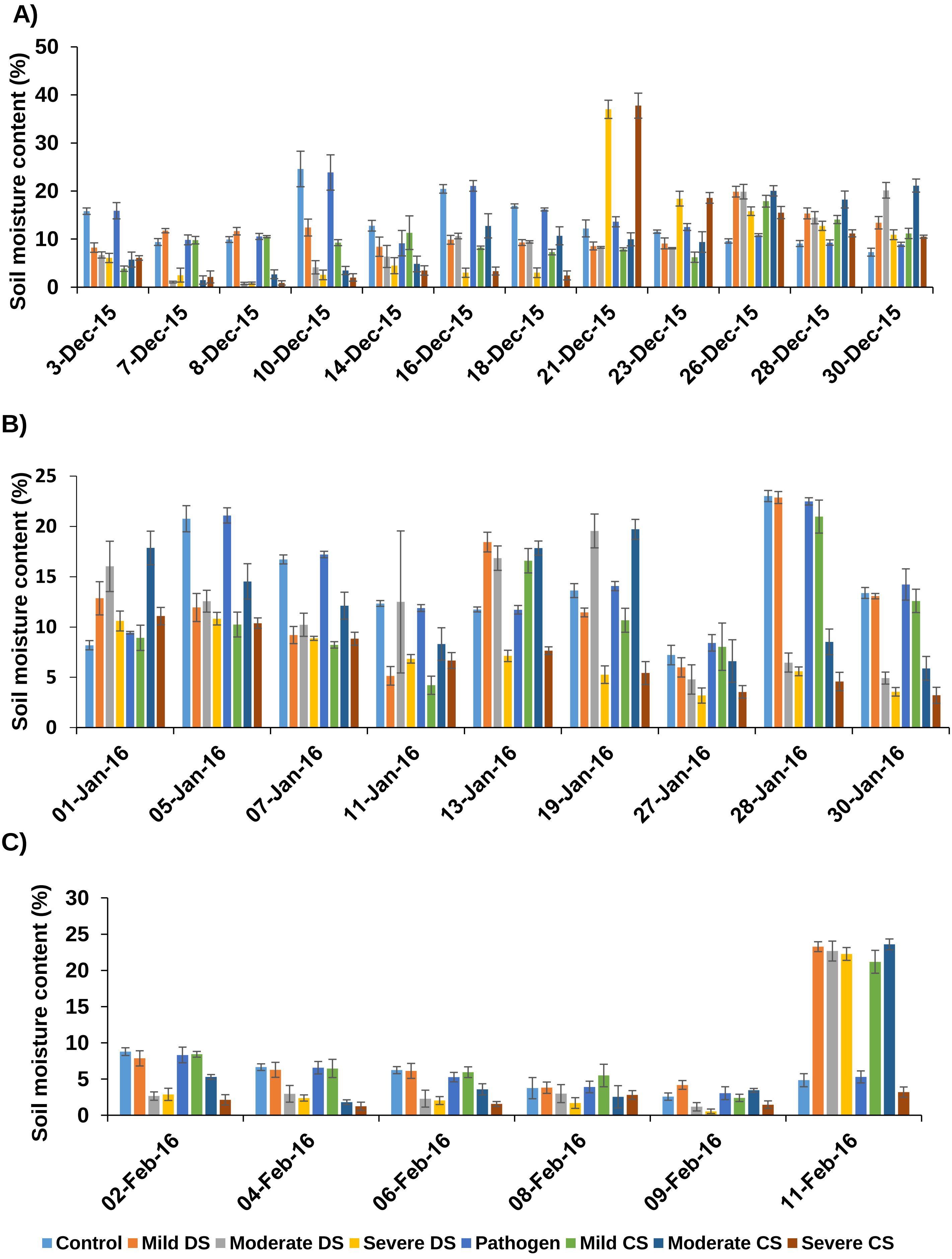

Supplementary Fig. S6. Relative soil moisture content of field location 1 in the year 2015-16. The graph represents soil moisture content (SMC) over three months December (A), January (B) and February (C) for the year 2015-16. SMC was recorded by Lutron PMS-714 soil moisture meter at 15 cm depth from the surface. Each bar is average of 3-4 block replicates with SEM. Three soil moisture readings were taken for each plot. Data for the soil moisture content along with SEM is provided in supplementary file S1. RCBD ANOVA with LSD post-hoc test was used for statistical analysis. Significant difference between means at $p<0.05$ are represented as different letter in supplementary file S1. Weather data for the respective day is provided in supplementary file S2. Details of the soil type and characteristics are provided in supplementary table S1.

Supplementary Fig. S7

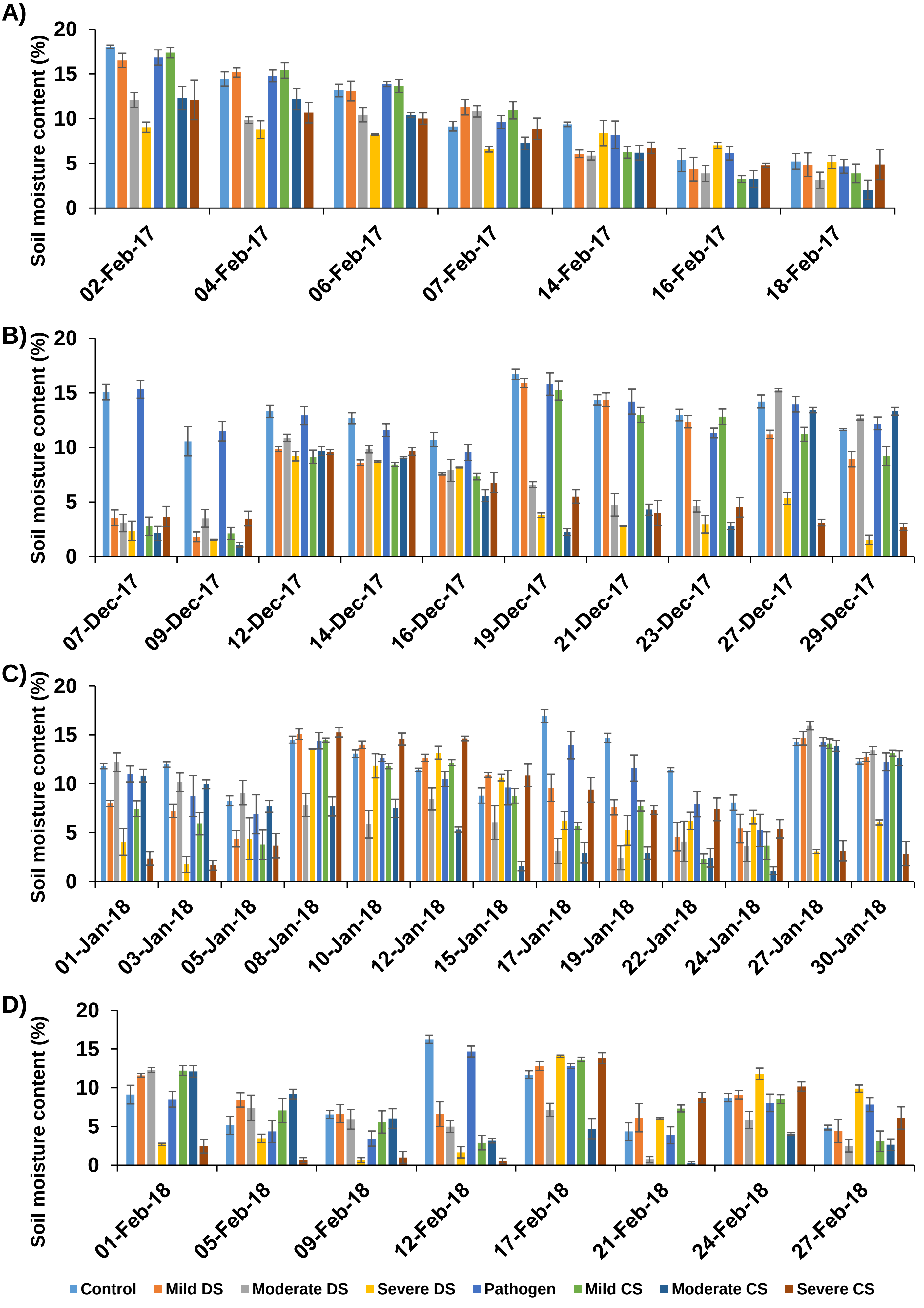

Supplementary Fig. S7. Relative soil moisture content of field location 1 in the year 2016-17 and 2017-18. The graph represents soil moisture content (SMC) for February 2017 (A) and for December (B), January (C) and February (D) 2017-18. SMC was recorded by Lutron PMS-714 soil moisture meter at 15 cm depth from the surface. Each bar is average of 3-4 block replicates with SEM. Soil moisture reading was taken from three different parts in each plot. Data for the soil moisture content along with SEM and statistics are given in supplementary file S1. RCBD ANOVA with LSD post-hoc test was used for statistical analysis. Significant difference between means at $p<0.05$ are represented as different letter in supplementary file S1. Weather data for the respective day is provided in supplementary file S2. Details of the soil type and characteristics are provided in supplementary table S1.

Supplementary Fig. S8

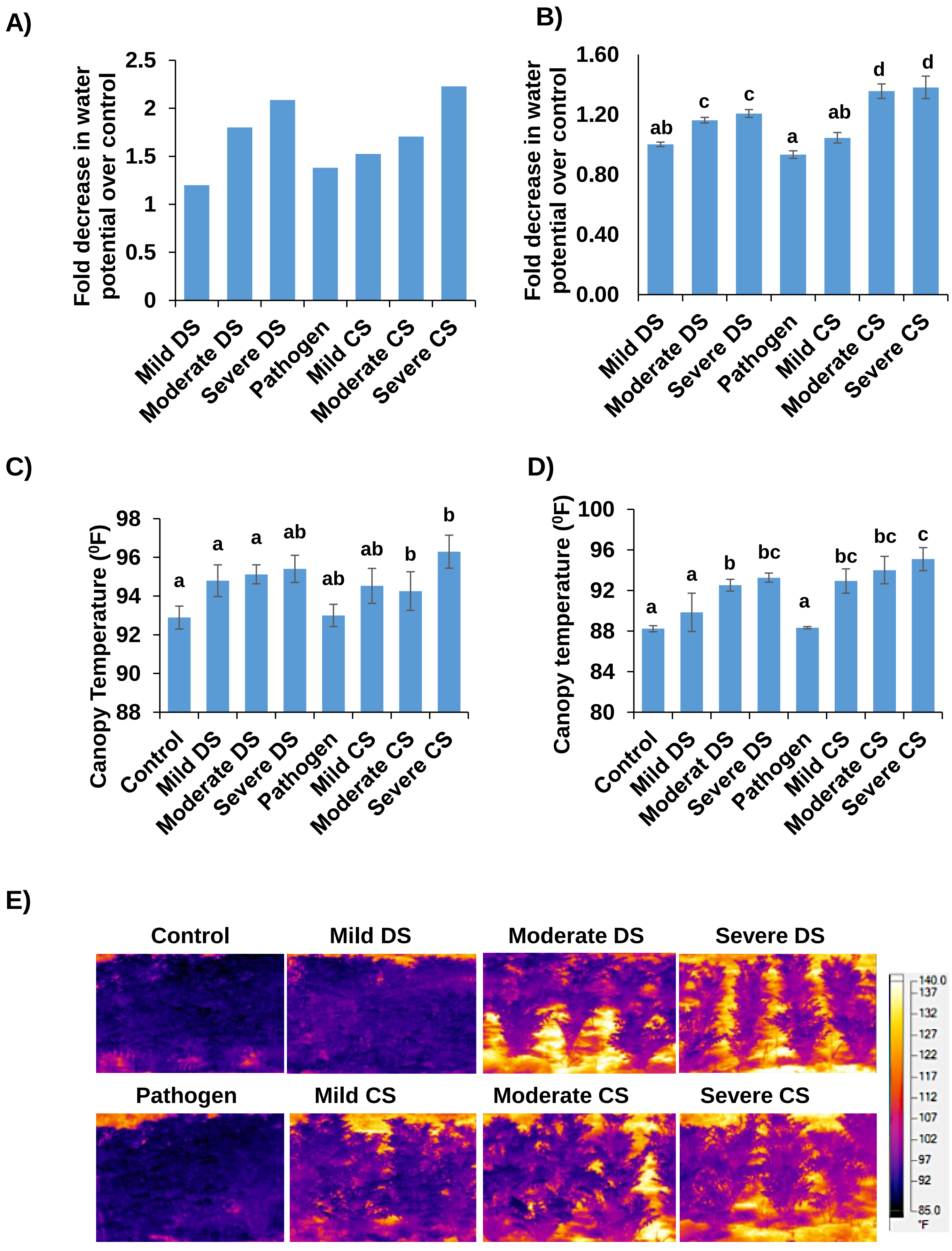

Supplementary Fig. S8. Estimation of drought stress by soil water potential and canopy temperature in field location 2. Drought stress in field location 2 experimental plots was estimated by soil water potential in the year 2015-16 (A) and 2017-18 (B) and also by canopy temperature in 2015-16 (C) and 2017-18 (D). Graph (A) and (B) represents fold decrease in water potential values over control. Water potential was measured as –MPa (megapascal) from one block replicate for each treatment in year 2015-16 (no statistical analysis) and three block replicates for each treatment during 2017-18 (see supplementary file S4). Water potential for year 2015-16 was measured in month of March and for year 2017-18 in month of February. Infrared images of chickpea canopy captured by Fluke Infrared Thermometer is shown in Figure S8E. Scale bar in 8E represents the temperature corresponding to the purple and orange color. Thermal image was taken for each treatment plot at around 11 am (images are not scalable) and the image was processed using SmartView 4.1 (Infrared Camera Analysis and Reporting Software, Fluke Corporation, Bangalore, India). The average temperature for 30-35 spots in each plot (excluding the bright yellow soil thermal image) recorded from IR images is represented in Figure S8C and S8D for the year 2015-16 and 2017-18 respectively. Each bar in Figure (B-D) is the average of three block replicates with SEM. RCBD Two way ANOVA was used to test the significant difference between the means. LSD All-Pairwise Comparisons test was used for multiple comparisons of mean. Different letters represent a significant difference at $p<0.05$. Weather data for the respective day is provided in supplementary file S2. Details of the soil information are provided in supplementary table S1.

Supplementary Fig. S9

Name of disease and causal agent	Infected plant showing foliar symptoms	Infected plant showing root symptoms	Respective fungi isolated from infected plants
**A)** Control (Uninfected)	i.	ii.	iii.
**B)** Dry root rot (*Rhizoctonia bataticola*)	i.	ii.	iii.
**C)** Black root rot (*Fusarium solani*)	i.	ii.	iii.
**D)** Fusarium wilt (*Fusarium oxysporum*)	i.	ii.	iii.
**E)** Collar rot (*Sclerotium rolfsii)*	i.	ii.	iii.

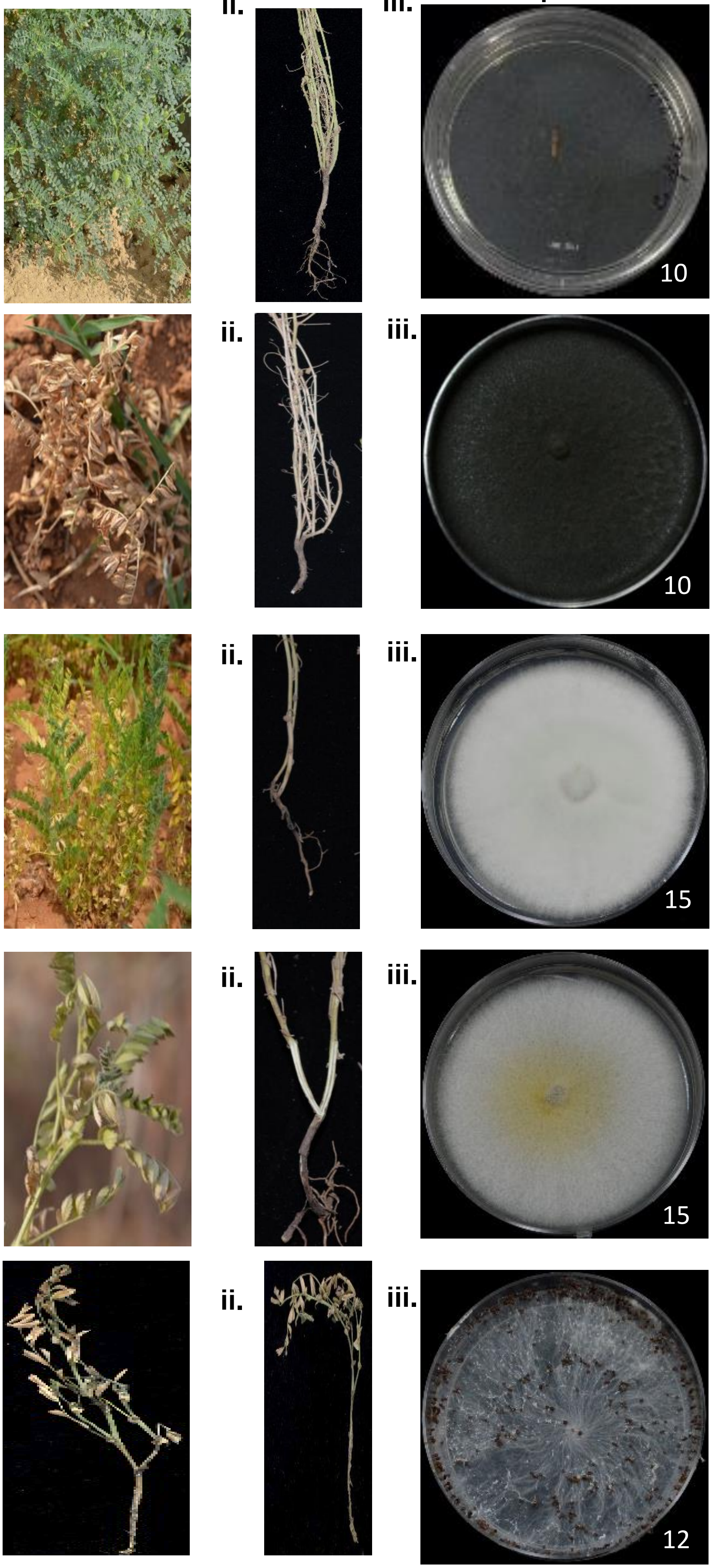

Supplementary Fig. S9

	Name of disease and causal agent		Infected plant showing foliar symptoms
**F)**	Botrytis gray mold (*Botrytis cinerea*)	**i.**	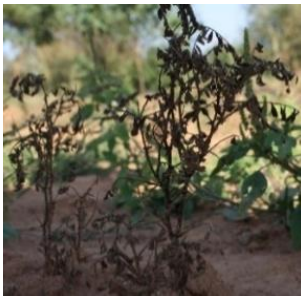
**G)**	Sclerotinia stem rot (*Sclerotinia sclerotiorum*)	**i.**	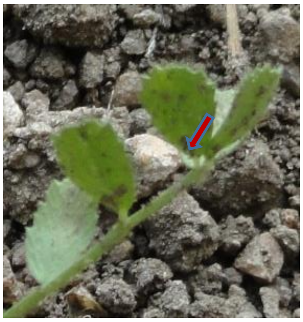
**H)**	*Beet western yellows virus (BWYV)*	**i.**	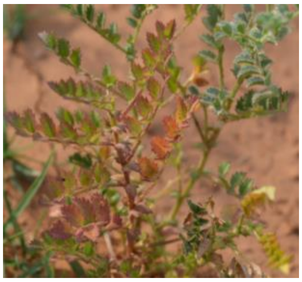

I)

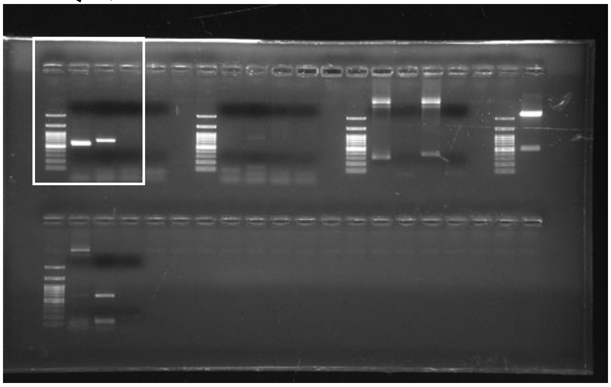

J)

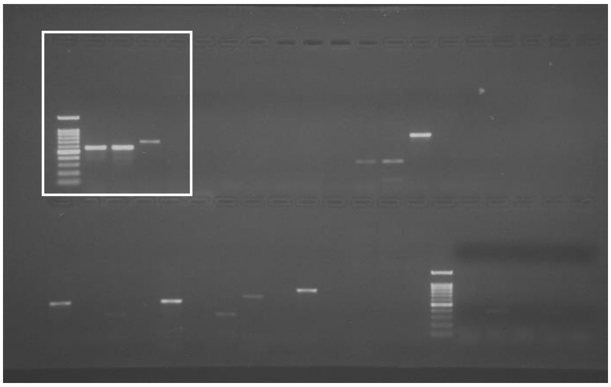

Supplementary Fig. S9. List of diseases encountered in the field experiment. Predominantly, the incidence of dry root rot (B) and black root rot (C) were high in chickpea in this field experiment. Diseases like fusarium wilt (D), collar rot (E), Botrytis gray mold (F), Sclerotinia stem rot (G) and *Beet western yellows virus* (BWYV) (I) were also observed. Here, we represent morphological features of control (A) and all diseased plants (i), root morphology of control and root rots (ii) and colony morphology of disease-causing fungi (iii). Diseased plants were identified with the foliar symptoms (i) and then uprooted to check the disease symptoms in the root (ii). A small part of surface sterilized fungal infected root tissue was cultured on PDA media for fungal identification. The number at the bottom of the plate represents the age of the culture (iii). Further, genomic DNA was isolated from the fungal plate using DNAzol® Reagent, and ITS sequence (~600bp) was amplified by using universal ITS primers (ITS1, 5' TCCGTAGGTGAACCTGCGG 3' and ITS4, 5'TCCTCCGCTTATTGATATGC 3'); L, 100bp ladder; *R. bataticola*; *Sclerotium rolfsii*; N, negative control (no template) (I) and L, 100bp ladder; *F. oxysporum*; *F. solani*; *S. rolfsii*; N, negative control (no template) (J). The bands corresponding to these figures are outlined with white border (I & J) Pictures are meant to show disease symptoms and not to the scale.

Supplementary Fig. S10

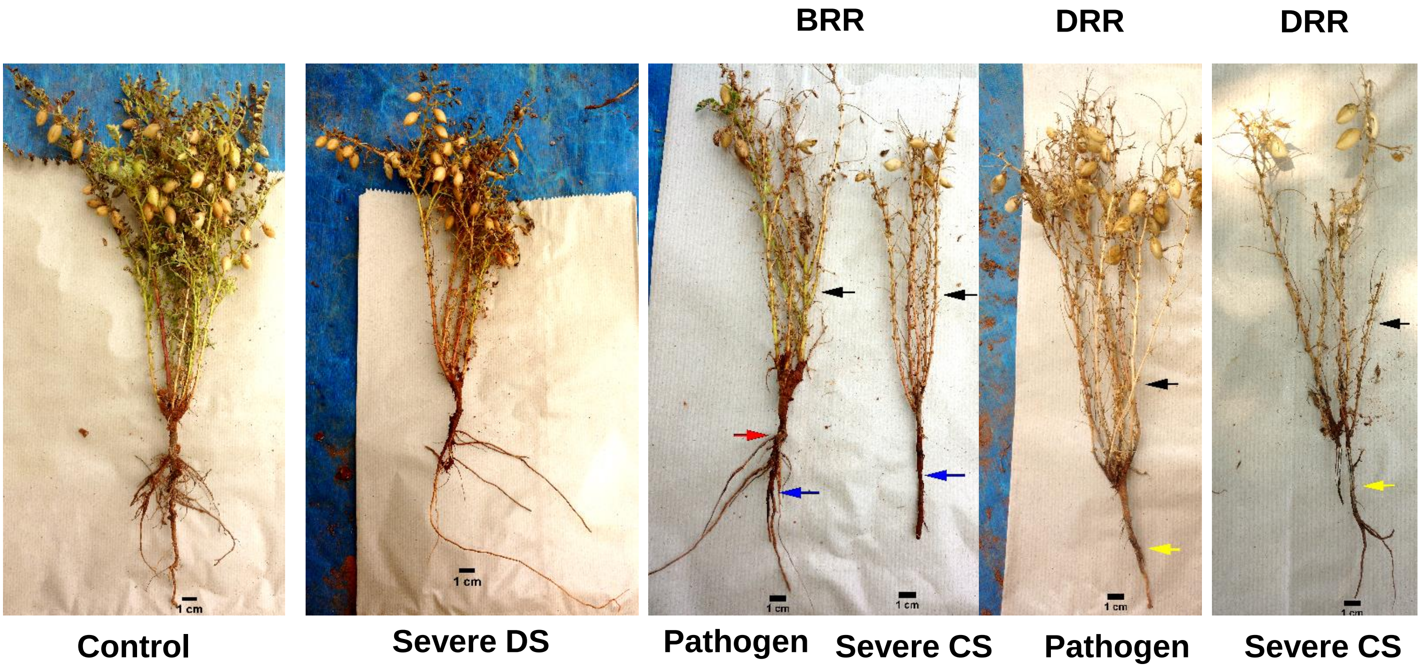

Supplementary Fig. S10. The morpho-physiological response of plants subjected to combined stress in field. In a field experiment, chickpea variety, PUSA 372, was subjected to seven different stress treatments viz., control, mild drought, moderate drought, severe drought, pathogen, mild combined stress, moderate combined stress and severe combined stress and control. Plants were examined for disease symptoms at three months after sowing. Disease symptoms such as reduction in foliar mass (black arrow), lateral root number (red arrow) and root blackening (blue arrow) and dried root (yellow arrow). Reduction in foliar mass and lateral root number was observed in severe drought (DS), pathogen (BRR), severe combined stress (CS) (BRR), pathogen (DRR) and severe CS (DRR). These images were captured in field location-2 in the year 2018. These images are representative plant symptoms from different treatments.

Supplementary Fig. S11

A)

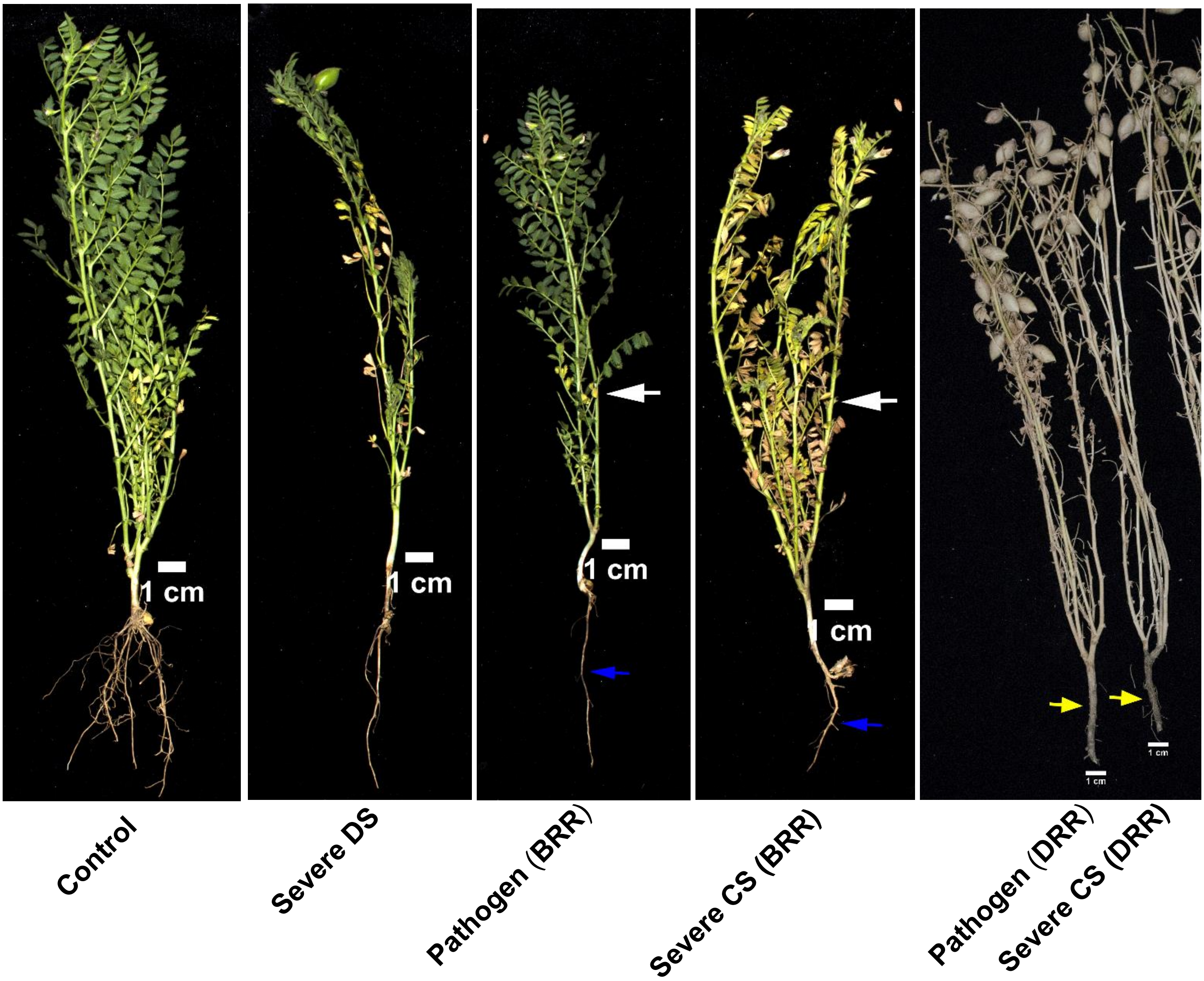

Supplementary Fig. S11. The severity of BRR and DRR diseases in field experiment. In a field experiment, chickpea variety, Pusa 372, was subjected to different stress treatments (Supplementary fig. S2). Plants were examined for disease symptoms (A). Disease symptoms such as reduction in foliar mass (white arrow), lateral root number (blue arrow) and root blackening (yellow arrow) and dried root (yellow arrow) were observed. Reduction in foliar mass (A) and lateral root number (A) was observed in severe DS, pathogen (BRR), combined CS (BRR), pathogen (DRR) and CS (DRR) compared to control. These images were captured in field location-1 in the year 2018. These images are representative plant symptoms from different treatments. Control, severe DS, pathogen (BRR), combined CS (BRR) were recorded 2 months after sowing and pathogen (DRR) and combined CS (DRR) was recorded 3 months after sowing.

Supplementary Fig. S12

A)

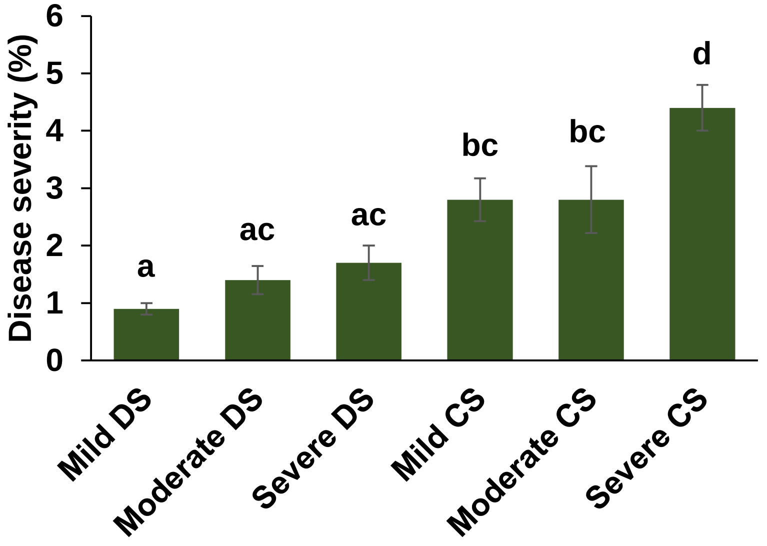

Supplementary Figure S12. Disease severity of Sclerotinia stem rot in chickpea under drought and combined stress treatments in field location-3. Disease severity for Sclerotinia stem rot in chickpea var. 372 was studied in different drought and combined stress treatments from field location 3. Disease severity was divided into five stages from 1-5 (A) based on disease symptoms in the plant. Score 0 = no black irregular spot, Score 1= black irregular spot in one branch of chickpea plant, Score 2= black irregular spot in two branches of chickpea plant, Score 3= disease in three branches of chickpea plant, Score 4= disease in four branches of chickpea plant, Score 5= disease in whole plant, Disease severity was calculated from at least 5 plants in a single treatment plot. Each bar represents the average of disease severity from 3 block replication plots with SEM. One way ANOVA was used to test the significant difference between the means. Tukey's pos-thoc test was used for multiple comparisons between means. Different letters represent a significant difference at $p<0.05$.

Supplementary Fig. S13

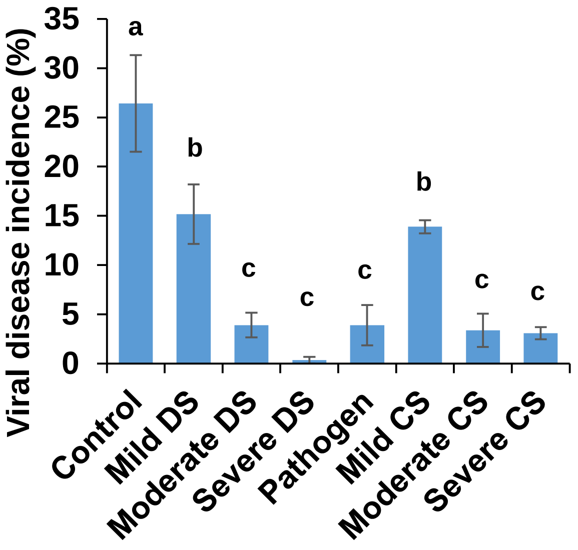

Supplementary Figure S13. Effect of drought stress on *Beet Western Yellow Virus* like disease incidence in field location 2. Viral disease was identified based on symptoms showed in supplementary fig. S9. Percent disease incidence (DI) per treatment plot was calculated based on symptoms and average of DI of 4 block replicates with SEM are represented in the graph. One way ANOVA was used to test the significant difference between the means. Tukey's post-hoc test was used for multiple comparisons between means. Different letters represent a significant difference between mean at $p<0.05$.

Supplementary Fig. S14

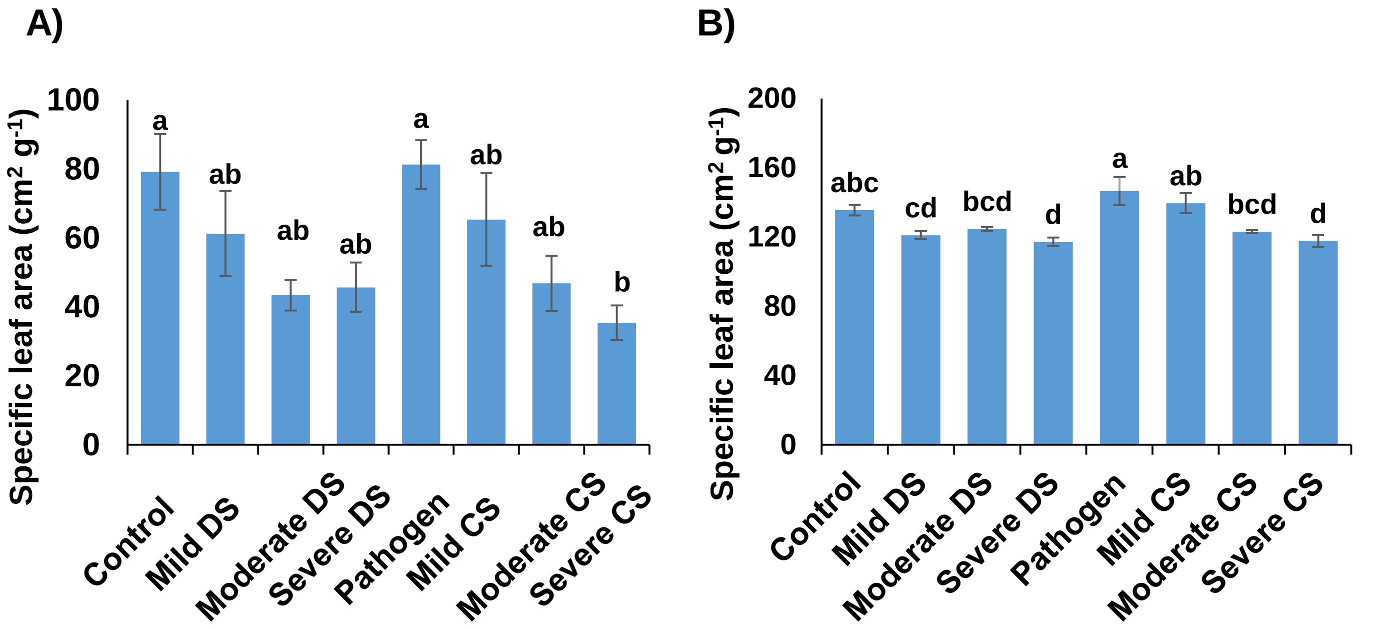

Supplementary Figure S14. Impact of drought stress and combined stress treatments on specific leaf area. Graph (A) and (B) represents a specific leaf area (SLA) for field location-1 and field location-2 respectively. Specific leaf area is calculated as ratio of leaf area (cm^2) to dry weight (gm). We observed difference in SLA between field location-1 and field location-2. this difference could be attributed to the edaphic (supplementary file S1) and environmental differences (supplementary file S2) in the two locations. Each bar represents the average of 3-4 block replicates with SEM. SLA was recorded from three different points within treatments plots. RCBD ANOVA with Tukey's post-hoc test was used to test the significant difference between the means. Different letters represent a significant difference between means at $p<0.05$.

Supplementary Fig. S15

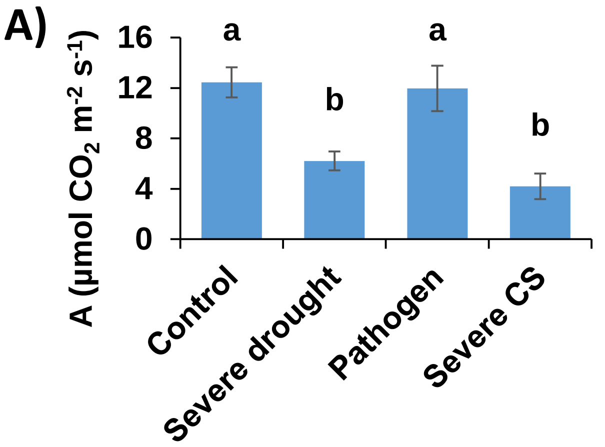

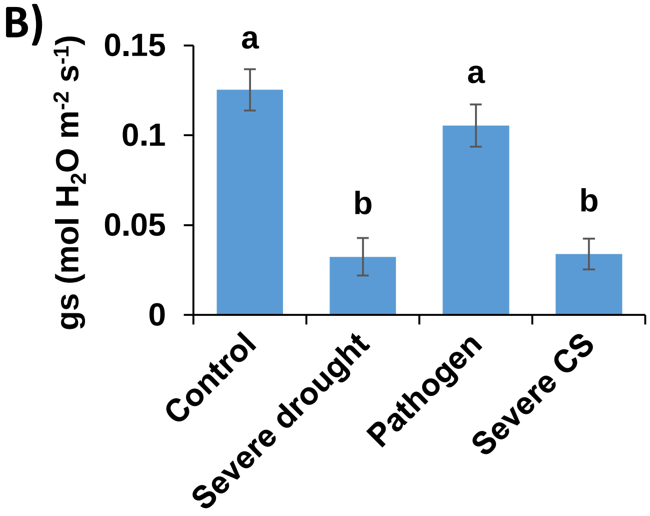

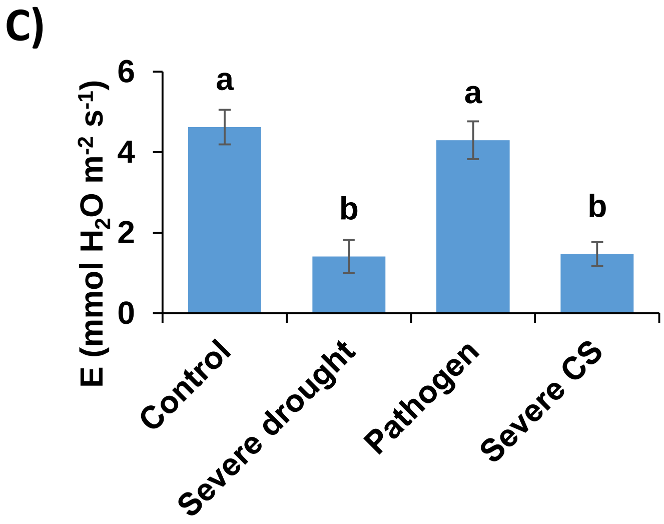

D)

		Pot experiment	
		BRR	DRR
	Photosynthetic rate (A)	0.76	0.99*
Field experiment	Stomatal conductance (GS)	0.99*	0.98*
	Transpiration rate (E)	0.98*	0.99*

Supplementary Fig. S15. Impact of combined drought and pathogen infection on leaf gas exchange parameters at field location-1. Leaf gas exchange parameters such as photosynthetic rate (A), stomatal conductance (B) and transpiration rate (C) were measured in plants under treatments in field location-1 using LICOR-6400 XT. Three plants in each block replicate was measured and considered as technical replicate. Graphs represent average ±SEM from three block replicates. RCBD ANOVA with LSD All-Pairwise Comparisons was used for statistical analysis. Different letters above each column represent the significance difference in mean at $p<0.05$. Leaf gas exchange parameters from field and pot experiments were compared by Pearson's correlation (D). Asterisk (*) in table (D) represents significant at $p<0.01$. Three out of seven treatments such as severe drought, pathogen and severe combined stress and control were used to make this graph to show significant reduction in leaf gas exchange parameters.

Supplementary Fig. S16

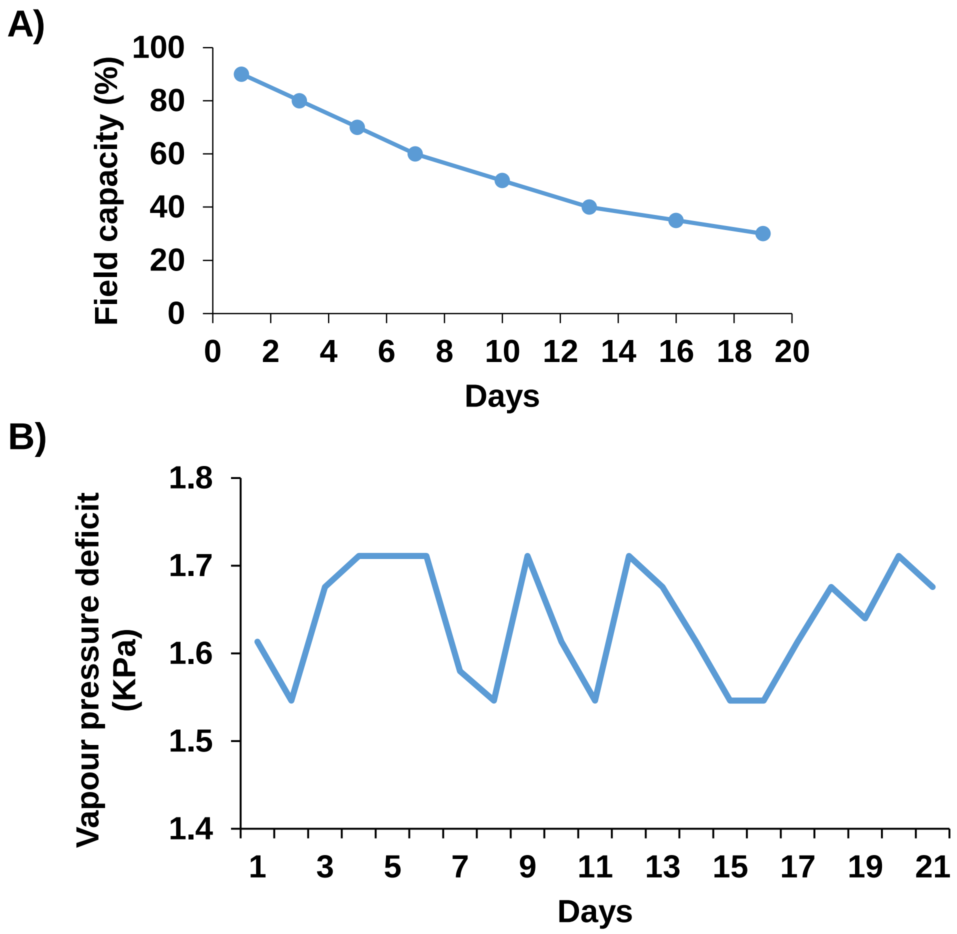

Supplementary Fig. S16. Standardization of drought stress imposition protocol and growth room conditions. Chickpea plants (genotype JG 62) for the pot combined stress experiment were grown in soilrite. We recorded the field capacity of soilrite every alternate day post-drought stress imposition to estimate the days required to achieve the 35% FC for moderate drought imposition. The drought was imposed by water-withholding and field capacity of the soilrite was measured gravimetrically as described by Gupta et al., 2016. The pot weight and dry soilrite weight (DW) were noted. The pot with soilrite was watered and allowed to absorb until a saturation point is achieved. Then, the pot was removed from the water and kept at room temperature overnight to allow the removal of excess trapped water and leaving only water absorbed and adsorbed to soilrite. Then, the saturation weight (SW) was noted. Field capacity was measured every day (A) using the following formula, Field capacity (FC)= [FW - DW)/(SW - DW)] × 100. FW=fresh weight. Desired drought level (35% FC) was achieved in 16 days (A). Vapor pressure deficit (VPD) influences the evapotranspiration and thus affects the rate of drought stress. We determined VPD throughout the experiment using the formula, VPD = ((100 - RH)/100)*SVP. SVP was taken from computed SVP value for a given temperature1 (B). SW=Saturated weight, AW=Actual weight, DW=dry weight, RH= relative humidity, SVP= saturated vapor pressure, VPD= vapor pressure deficit.

Reference:

1. Murray, F. W. (1967). On the Computation of Saturation Vapor Pressure. *Journal of Applied Meteorology*, *6*, 203-204.
2. Gupta A., Dixit S.K. & Senthil-Kumar M. (2016). Drought stress predominantly endures *Arabidopsis thaliana* to *Pseudomonas syringae* infection. Frontiers in Plant Science, 7, 1-12.

Supplementary Fig. S17

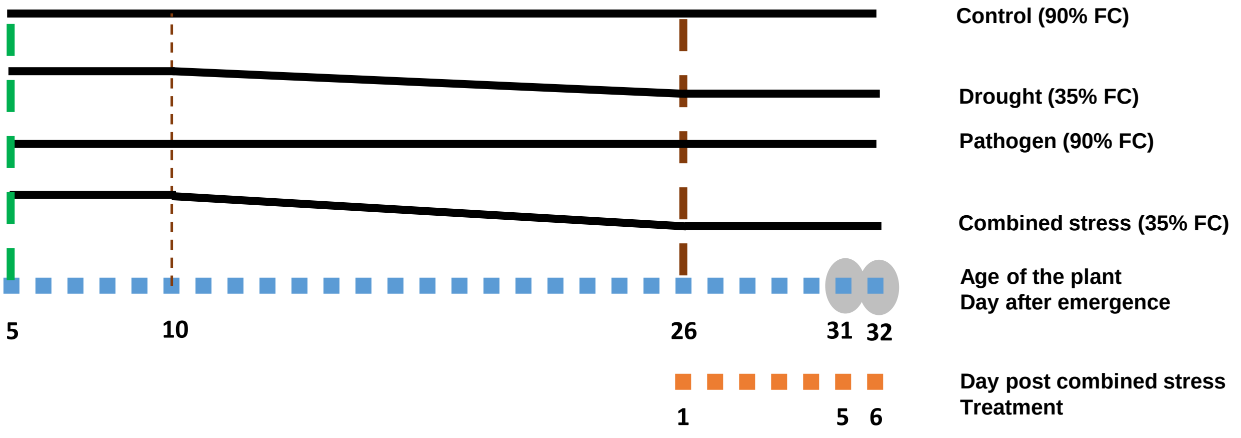

Supplementary Fig. S17. Outline of pot experiment with combined drought and *F. solani* in chickpea genotype, JG 62. A pot experiment with three treatments, control, pathogen only (pathogen), a drought only (drought) and combined drought and *F. solani* (combined stress) was performed to study the effect of drought stress on BRR disease progression in chickpea genotype JG62. Surface sterilized chickpea seeds were used in the experiment. Chickpea nursery was developed by sowing surface sterilized seeds and a five days old plants were uprooted and washed with sterilized water. Plant roots were dip inoculated into *F. solani* spore suspension (1.1X10^5 spore per ml) for 4 hours for pathogen and combined stress treatments while plant roots were dipped in sterilized RO for the same duration for control and drought treatments. The experiment was conducted with ten chickpea plants (10 biological replicates) for each treatment. All plants were maintained at 90% field capacity (FC) for the first five days. Water withholding for drought imposition was started from fifth day post replantation for drought, and combined stress treatments and FC was determined by the gravimetric method described by Gupta et al., 2016. Control and pathogen treatments were maintained at 90% FC. Desired drought level (35% FC) was achieved in 16 days for drought and combined stress. The drought was maintained for the next five days by replenishing water lost water by evapotranspiration. Gas exchange parameters were measured for all the treatments on five days post combined stress treatments and samples were collected for relative water content and microscopy observations on six days post combined stress treatments. The blue colored dotted line indicates the age of plant after germination and orange colored dotted lines indicate the days post combined stress treatments. The green vertical line indicates the day of dip inoculation. Thin and thick vertical lines indicate ate the day of drought and combined stress imposition respectively. The solid line indicates the field capacity level of treatments and a dotted line indicate the days of the experiment.

Supplementary Fig. S18

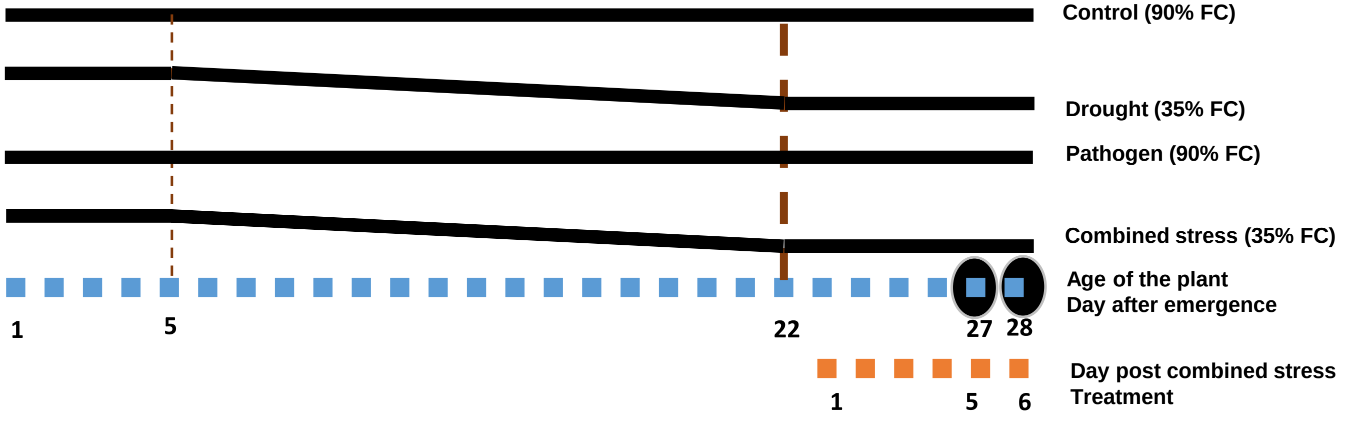

Supplementary Fig. S18. Outline of pot experiment with combined drought and *R. bataticola* in chickpea genotype, JG 62. A pot experiment with three treatments, control, pathogen only (pathogen), a drought only (drought) and combined drought and *R. bataticola* (combined stress) was performed to study the effect of drought stress on DRR disease progression in chickpea genotype JG 62. The experiment was conducted with ten chickpea plants (10 biological replicates) for each treatment. Surface sterilized chickpea seeds were used in the experiment. Pathogen and combined stress treatment were grown in a sick pot containing *R. bataticola* inoculum whereas, control and drought treatment were grown in autoclaved soilrite. All plants were maintained at 90% field capacity (FC) for the first five days. Drought stress was measured and maintained as described in supplementary fig. S16. Control and pathogen treatments were maintained at 90% FC. Desired drought level (35% FC) was achieved in 16 days for drought and combined stress. The drought was maintained for the next five days by replenishing water lost water by evapotranspiration. Gas exchange parameters were measured for all the treatments on fifth days post combined stress treatments, or 21st-day post-drought initiation and samples were collected for relative water content and microscopy observations on sixth day post combined stress. The blue colored dotted line indicates the age of plant after germination and orange colored dotted lines indicate the days post combined stress treatment. The thin vertical line indicates the start of drought imposition and the thick vertical line indicates the start of combined stress treatment. The solid line indicates the field capacity level of treatments and the dotted line indicate the days of the experiment.

Supplementary Fig. S19

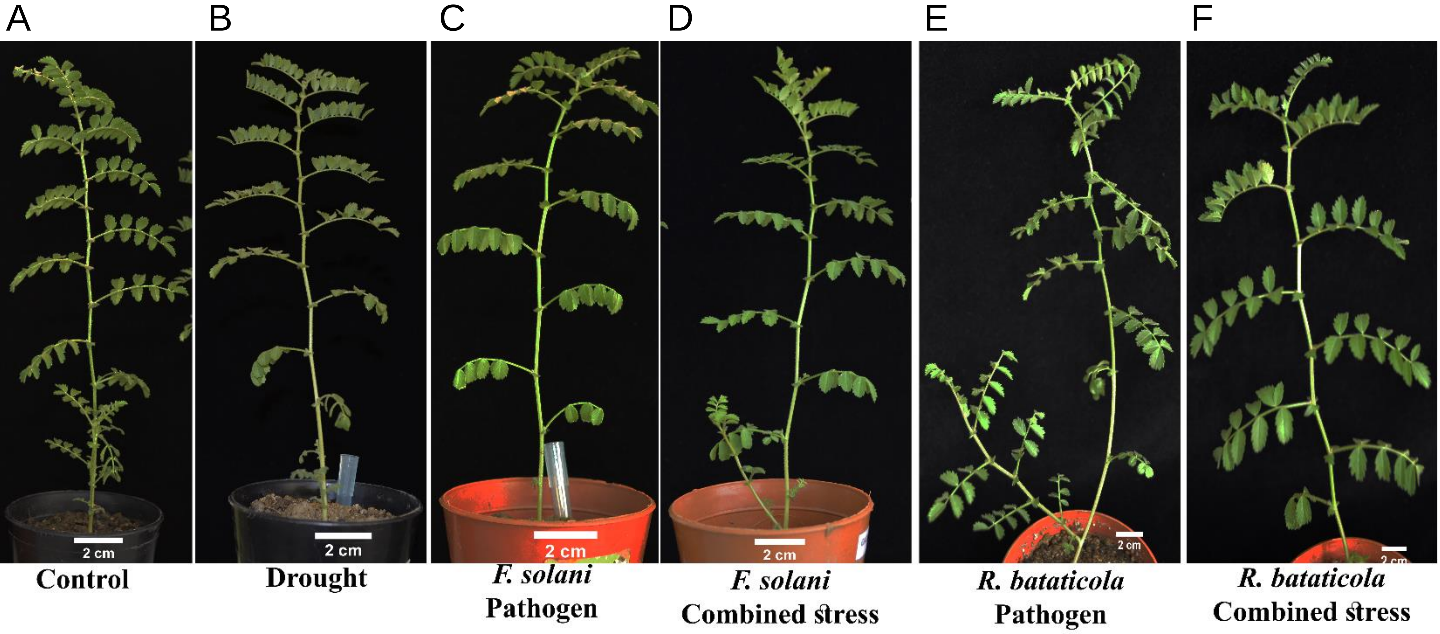

Supplementary Fig. S19: Physio-morphological study of chickpea PUSA372 under individual and combined drought and pathogen stress in pot experiment. Moderately resistant chickpea PUSA372 plants were imposed with only drought (B), only *F. solani* pathogen (C), only *R. bataticola* pathogen (E), combined drought and *F. solani* (D), and combined drought and *R. bataticola* pathogen (F). Plant physiology and disease incidence was checked after 22 days of stress imposition (Supplementary Fig. S17 & S18). The pathogen only and combined stress plants for both BRR and DRR did not show any significant disease symptoms 25 days post germination. Experiment was repeated trice. We conclude that moderately resistant genotype is resistant to pathogen under drought conditions too in pot experiments.

Supplementary Fig. S20

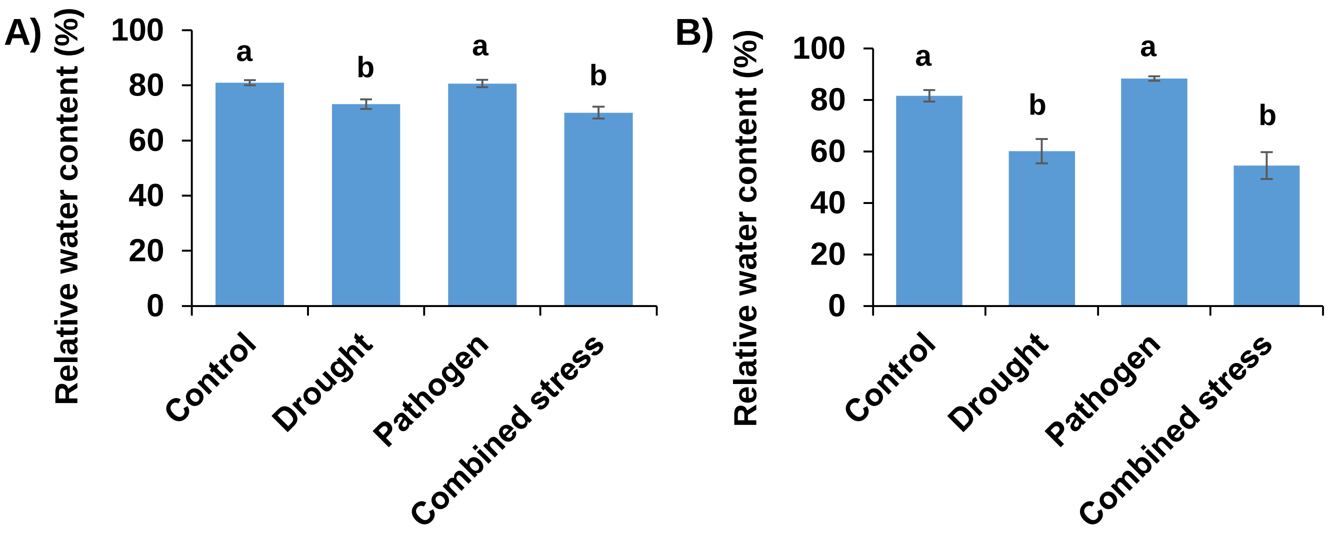

Supplementary Fig. S20. The relative water content of chickpea leaves under individual and combined stresses in a pot experiment. The relative water content of chickpea plants treated with individual and combined stress in pot experiments (supplementary figure S17, S18) was measured to assess the imposition of drought and combined stress. RWC was checked in the 4th leaf from six biological replicates and three technical replicates from each biological replicates. Drought and combined stress treatments displayed a significant reduction in leaf RWC compared to control and pathogen treatments in both, drought and *F. solani* combined stress experiment (A), and drought and *R. bataticola* combined stress experiment (B). Each bar represents the average of six biological replicates and three technical replicates of each biological replicate with SEM. One way ANOVA with Tukey's post-hoc test was used for mean comparison. Different letters represent a significant difference at $p<0.05$.

Supplementary Fig. S21

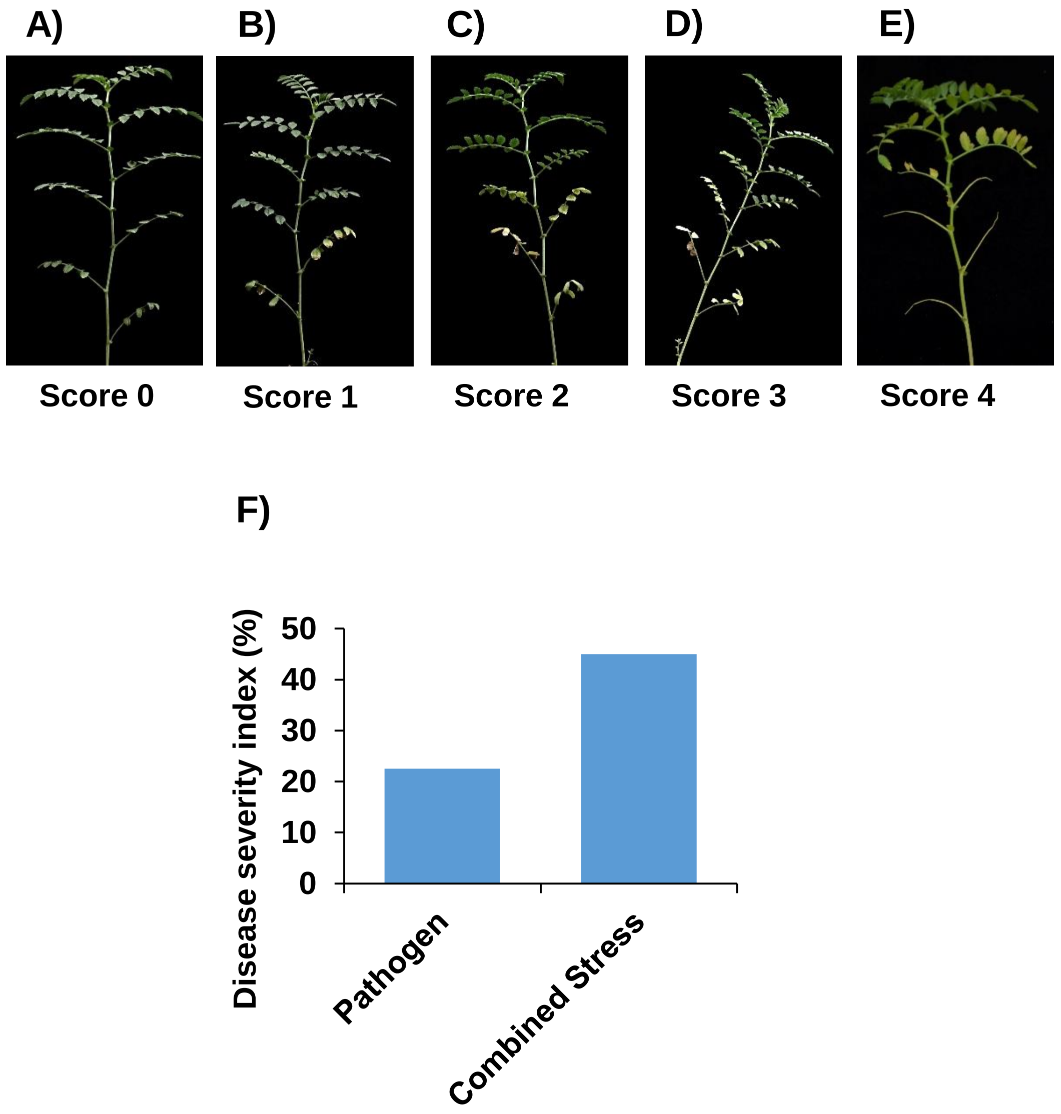

Supplementary Fig. S21. Disease severity index of black root rot disease under pathogen and combined stress in a pot experiment. Disease severity index (DSI) for black root rot under pathogen only and combined stress experiment was calculated based on foliar symptom on 21st-day post-drought stress treatment. Disease symptoms was divided into five scores for the calculation of DSI from 0-4 based on foliar symptoms in the plant. Score 0 = all green leaves (A), Score 1= two yellow leaves per chickpea plant (B), Score 2= four yellow leaves per chickpea plant (C), Score 3= six yellow leaves per chickpea plant (D), Score 4= eight yellow leaves or 8 shredded leaves per chickpea plant (E). DSI (F) was calculated from 10 biological replicates using formula, DSI (%) = [∑ (Class frequency × score of rating class)/(Total number of observations) × (maximal disease index)] × 100. DSI data is out of single experiment and therefore no statistical test was performed.

Supplementary Fig. S22

A)

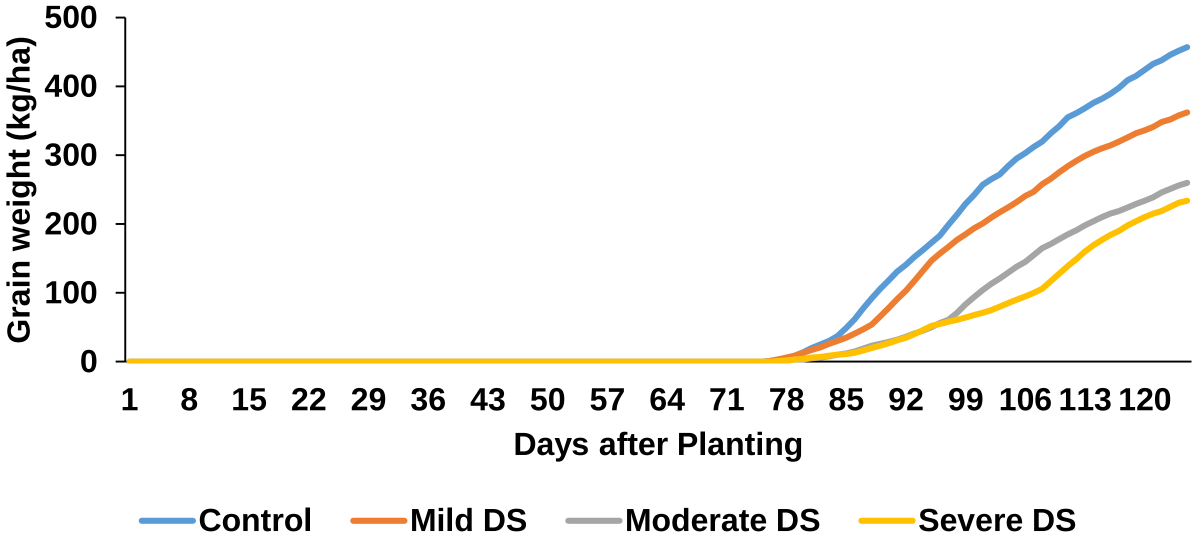

B)

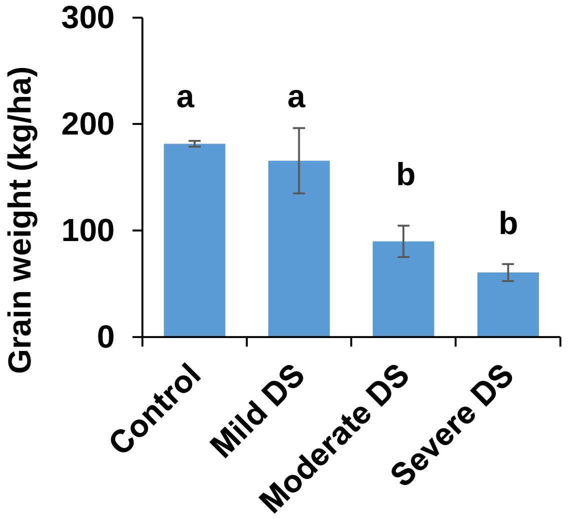

Supplementary Fig. S22. Yield under different irrigation regime from DSSAT crop simulation model and field experiment of field location 1 for the year 2015-16. DSSAT (The decision support system for agrotechnology transfer) is a crop simulation model for agrological predictions. We entered the details of soil characteristics (supplementary table S1), environmental conditions including temperature, PAR, rainfall (supplementary file S2), irrigation schedule (supplementary figure S2), area used for cultivation, and details of the fertilizer and fungicide used (supplementary figure S2) in DSSAT v4.7, chickpea module (https://dssat.net/) for simulation. The outcome of the DSSAT v4.7 model for grain yield (A) and actual grain yield for the same year in field location 1 (B) was represented here for comparison. The trend for the reduction in grain weight with the increase in drought stress is similar in both the graphs. For treatments control, mild DS, moderate DS and severe DS in graph (A), irrigation schedule corresponding to actual field experiment has been entered in DSSAT.

Supplementary Fig. S23

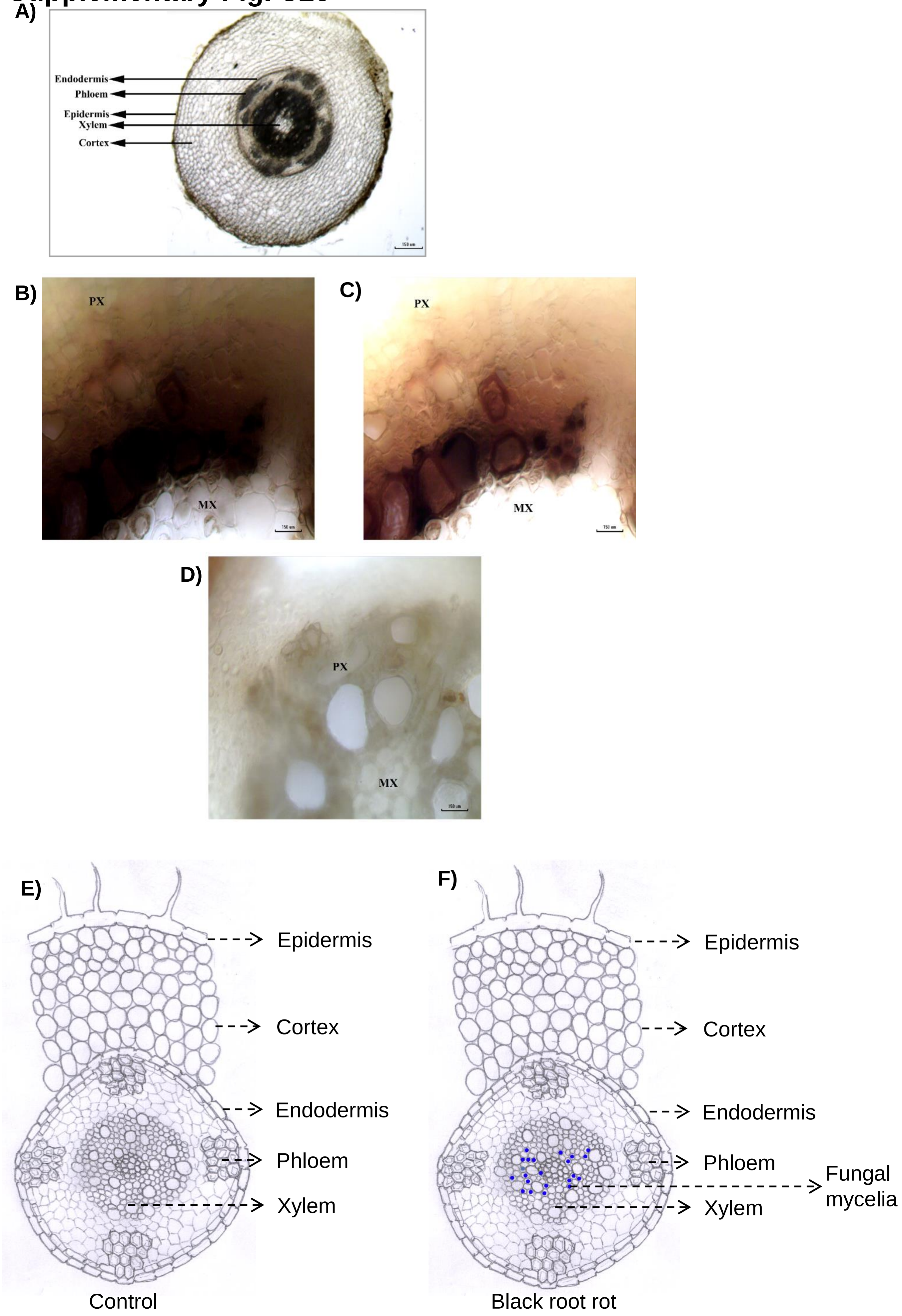

Supplementary Fig. S23. Microscopy images showing transverse section of chickpea root under combined stress. Transverse hand sections of plant roots from individual and combined stress treatments were cut and observed under 4X (A) and 40X objectives of LMI BM-X microscope. Epidermis, endodermis, cortex parenchyma and xylem and phloem are marked in root section captured at 4X objectives (A). Xylem regions of roots under combined drought and *F. solani* infection (B) and combined drought and *R. bataticola* infection (D) are shown. Image (C) is the brightened version of image (B). Root section were not stained and dark brown coloration in the root section is natural color developed in root after *F. solani* infection. MX, metaxylem; PX, protoxylem[1&2]. Scale bar represents 150 µm, three technical replicates used. All images are captured under white balanced background. Hand drawn root section of control (E) and black root rot (F) pictures are provided for the clarification.

Reference:

1. Castillo, P., Navas-Cortés, J. A., Landa, B. B., Jiménez-Díaz, R. M., & Vovlas, N. (2008). Plant-parasitic nematodes attacking chickpea and their in planta interactions with rhizobia and phytopathogenic fungi. *Plant Disease*, *92*(6), 840-853.
2. Purushothaman, R., Zaman-Allah, M., Mallikarjuna, N., Pannirselvam, R., Krishnamurthy, L., & Gowda, C. L. L. (2013). Root anatomical traits and their possible contribution to drought tolerance in grain legumes. *Plant Production Science*, *16*(1), 1-8.

Supplementary Table S1: Soil characteristics and nutrients status for field location 1 and 2.

Soil test report*			
	**2015-16**	**2016-17**	
**Parameters**	**Field location 1**	**Field location 1**	**Field location 2**
pH(1:2)	8.57	7.4	7.31
EC (μS/m) (1:2)	0.29	1.8	0.29
Organic C (%)	0.28	0.70	0.66
Water holding capacity (%)	40.7	NA	NA
Available nutrients (kg/ha)			
N	125	188	176
P	39.8	53.3	68.4
K	282	151	338
Soil characteristics			
Soil type	Loam	Loam	Sandy Clay
Soil texture			
Sand (%)	NA	36	46
Silt (%)	NA	43	15
Clay (%)	NA	21	39
CEC (me/100 g)	NA	16.8	13.8

*Soil sample was taken from 5 different sites at a depth of around 50 cm from each field location and mixed. 1:2 indicates ratio of soil and water. pH and EC (electrical conductivity) was tested in 1:2 ratio of soil and water. Organic C= organic carbon, N= nitrogen, P=phosphorus, K-potassium, CEC=cation exchange capacity, me= mini equivalents, NA= data not available. Soil testing was done at central laboratory for soil and plant analysis, division of soil science and agricultural chemistry, ICAR-Indian agricultural research institute, New Delhi. All soils are normal with low to medium organic carbon content.

Supplementary Table S2: Correlation matrix between BRR incidences for the field trial on assessing the impact of combined stress.[$]

		Field-1	Field-1	Field-2	Field-1	Field-2	Field-1	Field-2
		**2014-15**	**2015-16**	**2015-16**	**2016-17**	**2016-17**	**2017-18**	**2017-18**
**Field-1**	**2014-15**		0.89†	0.94‡	0.84†	0.86†	0.90†	0.80
**Field-1**	**2015-16**			0.95‡	0.96‡	0.79*	0.81*	0.85†
**Field-2**	**2015-16**				0.91†	0.75*	0.80*	0.73
**Field-1**	**2016-17**					0.83*	0.83*	0.88†
**Field-2**	**2016-17**						0.98‡	0.91†
**Field-1**	**2017-18**							0.89†
**Field-2**	**2017-18**							

[$] values in the table is Pearson's correlation (*r*). *, $p \leq 0.05$; †, $p \leq 0.01$; ‡ $p \leq 0.001$

≤

Supplementary Table S3: Correlation matrix between DRR incidences for the field trial on assessing the impact of combined stress.[$]

		Field-1	Field-2	Field-1	Field-2	Field-1	Field-2	Field-1	Field-2
		**2014-15**	**2014-15**	**2015-16**	**2015-16**	**2016-17**	**2016-17**	**2017-18**	**2017-18**
**Field-1**	**2014-15**		0.90*	0.82*	0.87†	0.72*	0.90†	0.77*	0.93‡
**Field-2**	**2014-15**			0.95*	0.99†	0.87	0.85	0.96†	0.99†
**Field-1**	**2015-16**				0.86†	0.65	0.93‡	0.82*	0.93‡
**Field-2**	**2015-16**					0.88†	0.89†	0.96‡	0.86†
**Field-1**	**2016-17**						0.77*	0.89†	0.60
**Field-2**	**2016-17**							0.80*	0.90†
**Field-1**	**2017-18**								0.79*
**Field-2**	**2017-18**								

[$] values in the table is Pearson's correlation (*r*). *, $p \leq 0.05$; † , $p \leq 0.01$; ‡ $p \leq 0.001$

Supplementary Table S4: Yield to yield correlation matrix for the field trial on assessing the impact of combined stress.$

		Field-1	Field-2	Field-1	Field-2	Field-1	Field-2
		**2015-16**	**2015-16**	**2016-17**	**2016-17**	**2017-18**	**2017-18**
**Field-1**	**2015-16**	1	0.96‡	0.93‡	0.92†	0.96‡	0.93‡
**Field-2**	**2015-16**		1	0.86†	0.82*	0.91†	0.85†
**Field-1**	**2016-17**			1	0.95‡	0.93‡	0.97‡
**Field-2**	**2016-17**				1	0.93‡	0.91†
**Field-1**	**2017-18**					1	0.94‡
**Field-2**	**2017-18**						1

$ values in the table is Pearson's correlation (*r*) *, *p* ≤0.05; † , *p* ≤0.01; ‡ *p* ≤0.001**.**

Supplementary Table S5: Correlation between yield and BRR incidence from the field trial on assessing the impact of combined stress.$

			Yield					
			**Field-1**	**Field-2**	**Field-1**	**Field-2**	**Field-1**	**Field-2**
			**2015-16**	**2015-16**	**2016-17**	**2016-17**	**2017-18**	**2017-18**
**BRR**	**Field-1**	**2015-16**	**-0.81***	-0.76*	-0.78*	-0.92*	-0.85†	-0.72*
	**Field-2**	**2015-16**	-0.72*	**-0.65**	-0.78*	-0.89†	-0.77*	-0.68
	**Field-1**	**2016-17**	-0.89†	-0.81*	**-0.84†**	-0.93‡	-0.92†	-0.79*
	**Field-2**	**2016-17**	-0.95‡	-0.91†	-0.98‡	**-0.93**‡	-0.95‡	-0.98‡
	**Field-1**	**2017-18**	-0.89†	-0.84†	-0.95‡	-0.91†	**-0.94‡**	-0.95‡
	**Field-2**	**2017-18**	-0.95‡	-0.96‡	-0.85†	-0.86†	-0.96‡	**-0.86†**

Values in the table is Pearson's correlation (*r*). *, *p* ≤0.05; † , *p* ≤0.01; ‡ *p* ≤0.001

Supplementary Table S6: Correlation between yield and DRR incidence from the field trial on assessing the impact of combined stress.$

			Yield					
DRR			**Field-1**	**Field-2**	**Field-1**	**Field-2**	**Field-1**	**Field-2**
			**2015-16**	**2015-16**	**2016-17**	**2016-17**	**2017-18**	**2017-18**
	**Field-1**	**2015-16**	**-0.77***	-0.70	-0.69	-0.83*	-0.83*	-0.76*
	**Field-2**	**2015-16**	-0.79*	**-0.72***	-0.77*	-0.90*	-0.83*	-0.74*
	**Field-1**	**2016-17**	-0.50	-0.47	**-0.49**	-0.65	-0.61	-0.43
	**Field-2**	**2016-17**	-0.76*	-0.70	-0.66	**-0.84***	-0.83*	-0.66
	**Field-1**	**2017-18**	-0.68	-0.64	-0.67	-0.78*	**-0.74***	-0.69
	**Field-2**	**2017-18**	-0.95‡	-0.90†	-0.86†	-0.92†	-0.96‡	**-0.89†**

$ values in the table is Pearson's correlation (*r*). *, *p* ≤0.05; † , *p* ≤0.01; ‡ *p* ≤0.001
